# Thirteen *Camellia* chloroplast genome sequences determined by high-throughput sequencing: genome structure and phylogenetic relationships

**DOI:** 10.1186/1471-2148-14-151

**Published:** 2014-07-07

**Authors:** Hui Huang, Chao Shi, Yuan Liu, Shu-Yan Mao, Li-Zhi Gao

**Affiliations:** 1 Plant Germplasm and Genomics Center, Germplasm Bank of Wild Species in Southwest China, Kunming Institute of Botany, Chinese Academy of Sciences, Kunming 650201, China; 2 University of the Chinese Academy of Sciences, Beijing 100039, China

**Keywords:** *Camellia*, Chloroplast genome, Phylogenetic relationships, Genomic structure, Taxonomic identification

## Abstract

**Background:**

*Camellia* is an economically and phylogenetically important genus in the family Theaceae. Owing to numerous hybridization and polyploidization, it is taxonomically and phylogenetically ranked as one of the most challengingly difficult taxa in plants. Sequence comparisons of chloroplast (cp) genomes are of great interest to provide a robust evidence for taxonomic studies, species identification and understanding mechanisms that underlie the evolution of the *Camellia* species.

**Results:**

The eight complete cp genomes and five draft cp genome sequences of *Camellia* species were determined using Illumina sequencing technology via a combined strategy of *de novo* and reference-guided assembly. The *Camellia* cp genomes exhibited typical circular structure that was rather conserved in genomic structure and the synteny of gene order. Differences of repeat sequences, simple sequence repeats, indels and substitutions were further examined among five complete cp genomes, representing a wide phylogenetic diversity in the genus. A total of fifteen molecular markers were identified with more than 1.5% sequence divergence that may be useful for further phylogenetic analysis and species identification of *Camellia*. Our results showed that, rather than functional constrains, it is the regional constraints that strongly affect sequence evolution of the cp genomes. In a substantial improvement over prior studies, evolutionary relationships of the section *Thea* were determined on basis of phylogenomic analyses of cp genome sequences.

**Conclusions:**

Despite a high degree of conservation between the *Camellia* cp genomes, sequence variation among species could still be detected, representing a wide phylogenetic diversity in the genus. Furthermore, phylogenomic analysis was conducted using 18 complete cp genomes and 5 draft cp genome sequences of *Camellia* species. Our results support Chang’s taxonomical treatment that *C. pubicosta* may be classified into sect. *Thea*, and indicate that taxonomical value of the number of ovaries should be reconsidered when classifying the *Camellia* species. The availability of these cp genomes provides valuable genetic information for accurately identifying species, clarifying taxonomy and reconstructing the phylogeny of the genus *Camellia*.

## Background

*Camellia*, comprising more than 200 species, is an economically and phylogenetically important genus in the family Theaceae [1]. Besides the abundance in phenotypic and species diversity, increasing attention has been paid to the genus, as they include several economically important members of their commercial and ornamental values. One of the most economic values of *Camellia* is the production of tea made from the young leaves of *C. sinensis* var. *sinensis* and *C. sinensis* var. *assamica* in the section *Thea*. The other most economically important species is *C. oleifera*, which has the longest history of cultivation and utilization in China for edible oil used primarily in cooking. Many other species of the genus *Camellia* were also used locally for seed oil production, such as *C. reticulata*[2]. Moreover, the *Camellia* species are of great ornamental values, particularly represented by *C. japonica*, *C. reticulata* and *C. sasanqua*.

As a result of frequent hybridization and polyploidization, *Camellia* is taxonomically and phylogenetically regarded as one of the most challengingly difficult taxa in plants. Traditional classification of species using a morphology-based system is often dynamic and unreliable, which is often affected by environmental factors. The lack of suitable DNA fragments and polymorphic genetic markers for phylogenic analysis have long obstructed the availability of a reliable phylogeny, adding the controversies about taxonomic classification that prevent us from better understanding the diversification and evolution of the genus *Camellia*. By using amplified fragment length polymorphisms (AFLPs) [3], simple sequence repeats (SSRs) [4], random amplified polymorphic DNA (RAPD) [5], inter-simple sequence repeat (ISSR) [6], internal transcribed spacer (ITS) [1,7] and several DNA loci [8], a number of previous studies gave further insights into the taxonomy and phylogeny of the *Camellia* species but still have not reached a satisfied resolution. A recent effort using whole chloroplast (cp) genome sequences of six *Camellia* species has generated useful data but still failed to determine their phylogenetic relationships, not agreeing with any taxonomic treatments [9].

The cp genomes could provide valuable information for taxonomic classification and the reconstruction of phylogeny as a result of sequence divergence between plant species and individuals. Owing to the absence of recombination and maternal transmission, the cp genomes are helpful for tracing source populations [10,11] and phylogenetic studies of higher plants for resolving complex evolutionary relationships [12-14]. It is particularly true for the case of *Camellia*, given its confusing phylogenetic relationships with large nuclear genomes [15]. Cp-derived markers, e.g. *rpl16* gene*, psbA-trnH, trnL-F* and *rpl32-trnL* intergenic spacer (IGS), were employed to study evolutionary relationships between tea plants [8,16]. Repetitive sequences within the cp genomes are also potentially useful for ecological and evolutionary studies of plants [17]. Not only will the information from cp genomes be useful for studying the taxonomy and phylogenetic relationships, but it will also facilitate cp transformation in the economically important plants. The next-generation sequencing techniques have revolutionized DNA sequencing via high-throughput capabilities but relatively low costs. As it is now more convenient to obtain cp genome sequences and promptly extend gene-based phylogenetics to phylogenomics.

In this study, we sequenced the 13 *Camellia* chloroplast genomes using next-generation Illumina genome analyzer platform. The sequenced *Camellia* species included up to 10 species and varieties (10/18) from sect. *Thea* with an emphasis of these species belonging to the section*.* Three representative species were additionally sampled, each from sect. *Camellia*, sect. *Corallina* and sect. *Archecamellia*, respectively. This study aims to examine global patterns of structural variation of the *Camellia* cp genomes and reconstruct phylogenetic relationships among the representative species. The complete cp genome sequences of *Camellia* reported here are prerequisite for classifying the ‘difficult taxa’ and modifying these important economic plants by chloroplast genetic engineering techniques.

## Results and discussion

### Chloroplast genome sequencing and assembly

Using Illumina genome analyzer platform, we sequenced cp genomes of seven species and three varieties from sect. *Thea*, and each from sect. *Archecamellia*, *Corallina* and *Camellia*, respectively (Table 1). The three cp genomes of *C. sinensis* var. *assamica*, *C. oleifera* and *C. taliensis*[18] were used in our study with a minor revision by the two following steps: 1) assembled and manually checked more carefully; 2) PCR were used with high fidelity polymerase to verify the sequences in the four junctions. And then the revised *C. sinensis* var. *assamica* was employed as a reference while assembling the 13 sequenced cp genomes. Illumina paired-end (2 × 100 bp) sequencing produced large data sets for individual species. 5,504,058 (*C. tachangensis*) to 111,673,521 (*C. sinensis* var. *sinensis*) paired-end reads were mapped to the reference cp genome of *C. sinensis* var. *assamica*, reaching 35 to 711 × coverage on average across these cp genomes. After *de novo* and reference-guided assembly as in [19] with minor modifications, we obtained eight complete cp genomes and five draft cp genomes (Table 2). The four junction regions for each resulting cp genome were validated by using PCR-based sequencing with four pairs of primers (Additional file 1: Table S1). These genome sequences were deposited into the GenBank under accession numbers (KJ806274-KJ806286) (Additional file 2: Table S2).

**Table 1:** **Information of the sequenced *Camellia *** **chloroplast genomes according Min’s taxonomic treatment**[2]

**Species**	**Subgenus**	**Section**	**Collection sites**
*Camellia crassicolumna* var. *crassicolumna*	*Thea*	*Thea*	TRI
*Camellia fangchengensis*	*Thea*	*Thea*	ICSG
*Camellia grandibracteata*	*Thea*	*Thea*	TRI
*Camellia kwangsiensis*	*Thea*	*Thea*	TRI
*Camellia leptophylla*	*Thea*	*Thea*	ICSG
*Camellia ptilophylla*	*Thea*	*Thea*	ICSG
*Camellia sinensis* var. *dehungensis*	*Thea*	*Thea*	TRI
*Camellia sinensis* var. *sinensis*	*Thea*	*Thea*	TRI
*Camellia sinensis* var*. pubilimba*	*Thea*	*Thea*	TRI
*Camellia tachangensis*	*Thea*	*Thea*	TRI
*Camellia petelotii*	*Thea*	*Archecamellia*	ICSG
*Camellia pubicosta*	*Thea*	*Corallina*	ICSG
*Camellia reticulata*	*Camellia*	*Camellia*	KIB

TRI, Tea Research Institute, Yunnan Academy of Agricultural Science (Menghai, Yunnan, China);

ICSG, International *Camellia* Species Garden (Jinhua, Zhejiang, China);

KIB, Kunming Institute of Botany, Chinese Academy of Sciences (Kunming, Yunnan, China).

**Table 2:** The sequenced chloroplast genome features

**Complete genomes**	**Matched reads (bp)**	**Genome size (bp)**	**Mean coverage**	**LSC length (bp)**	**SSC length (bp)**	**IR length (bp)**	**GC content (%)**
*C. grandibracteata*	24,127,775	157,127	154	86,657	18,286	26,092	37.29
*C. leptophylla*	26,635,918	157,102	170	86,648	18,276	26,089	37.30
*C. sinensis* var. *dehungensis*	24,978,790	157,110	159	86,656	18,276	26,089	37.30
*C. sinensis* var. *sinensis*	111,673,521	157,117	711	86,663	18,276	26,089	37.29
*C. sinensis* var. *pubilimba*	7,753,104	157,086	49	86,679	18,267	26,096	37.30
*C. petelotii*	9,358,318	157,121	60	86,660	18,283	26,089	37.29
*C. pubicosta*	36,142,305	157,076	230	86,650	18,280	26,073	37.30
*C. reticulata*	56,357,778	156,971	359	86,606	18,235	26,065	37.30
*C. oleifera*	8,162,492	157,145	52	86,676	18,291	26,089	37.28
*C. sinensis* var. *assamica*	2,828,916	157,121	18	86,651	18,286	26,092	37.29
*C. taliensis*	2,828,754	157,087	18	86,650	18,287	26,075	37.29
**Incomplete genomes**	**Matched reads (bp)**	**Predicted genome size (bp)**	**Mean coverage**	**Number of gaps**	**Gap length (bp)**		
*C. crassicolumna* var. *crassicolumna*	6,595,133	157,100	42	180	40,530		
*C. fangchengensis*	39,446,507	157,364	251	138	30,491		
*C. kwangsiensis*	8,553,876	156,992	54	207	36,424		
*C. ptilophylla*	8,426,943	157,057	54	148	22,817		
*C. tachangensis*	5,504,058	157,009	35	345	57,250		

### Conservation of *Camellia* chloroplast genomes

**Figure 1:**
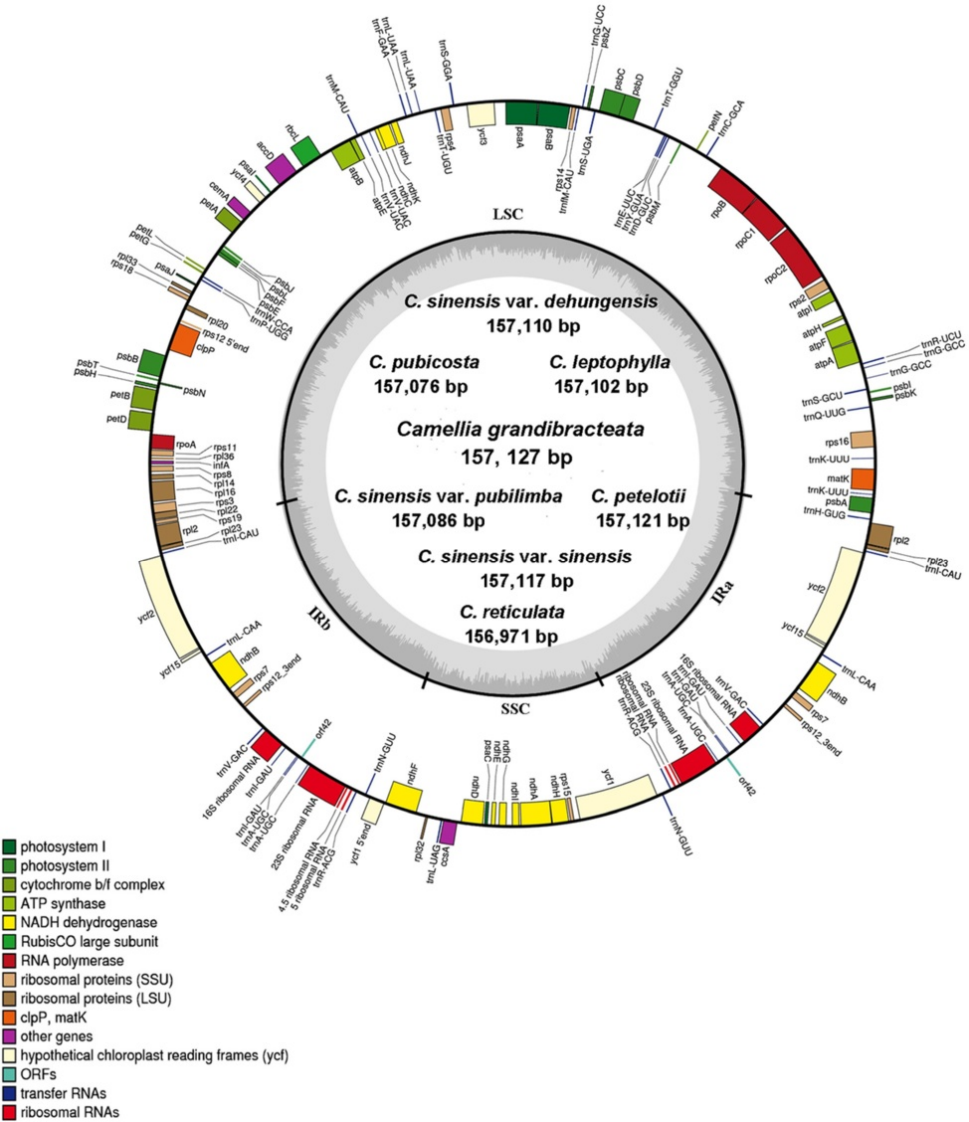
**Gene map of the *Camellia *chloroplast genomes.** Genes shown outside the outer circle are transcribed clockwise and those inside are transcribed counterclockwise. Genes belonging to different functional groups are color-coded. Dashed area in the inner circle indicates the GC content of the chloroplast genome.

All eight complete *Camellia* cp genomes were composed of single circular double-stranded DNA molecules. They displayed the typical quadripartite structure of most angiosperms, including the large single copy (LSC), the small single copy (SSC) and a pair of inverted repeats (IRa and IRb). There were no obvious sequence inversions or genomic rearrangements (Figure 1). Among these cp genomes, genome size ranged from 156,971 bp (*C. reticulata*) to 157,127 bp (*C. grandibracteata*). The length varied from 86,606 bp (*C. reticulata*) to 86,679 bp (*C. sinensis* var. *pubilimba*) in the LSC region, from 18,235 bp (*C. reticulata*) to 18,286 bp (*C. grandibracteata*) in the SSC region, and from 26,065 bp (*C. reticulata*) to 26,096 bp (*C. sinensis* var. *pubilimba*) in IR region. Each cp genome was found to harbor a total of 131 genes, including 86 protein-coding genes, 37 transfer RNA (tRNA) genes, and eight ribosomal RNA (rRNA) genes (Table 3). Of them, we identified 13 protein-coding genes, 14 tRNA coding genes and eight rRNA coding genes that are located within IRs. The LSC region contained 61 protein-coding and 22 tRNA genes, while the SSC region had 11 protein-coding and one tRNA gene. The *rps12* gene is an uniquely divided gene with the 5′ end exon located in the LSC region while two copies of 3′ end exon and intron are located in the IRs. The *ycf1* is located in the boundary regions between IRa/SSC, leading to incomplete duplication of the gene within IRs. There were 18 intron-containing genes, including six tRNA genes and 12 protein-coding genes, almost all of which are single-intron genes except for *ycf3* and *clpP*, each having two introns (Table 3). *matK* was located within the intron of *trnK-UUU* with the largest intron (2,487 bp). It was found that *ycf1*, *accD, rpl23* and *ycf2* are often absent in plants [20], but they were detected in the reported *Camellia* cp genomes in this study. Similar to other higher plants, one pair of genes, *atpB-atpE*, was observed to overlap each other with 3-bp. However, *psbC-psbD* had a 52-bp overlapping region in the *Camellia* cp genomes, which was observed an overlapping 53-bp in *Gossypium*[21]. Note that coding and non-coding regions account for 44.6% and 55.4% of the whole cp genome, respectively. The overall GC content was approximately 37.3%, which is almost identical with each other among the eight complete *Camellia* cp genomes.

**Table 3:** **Genes contained in the sequenced *Camellia *** **chloroplast genomes**

**Category**	**Group of genes**	**Name of genes**
Self replication	Large subunit of ribosomal proteins	rpl2^b,c^, 14, 16^b^, 20, 22, 23^c^, 32, 33, 36
	Small subunit of ribosomal proteins	rps2, 3, 4, 7^c^, 8, 11, 12^b-d^, 14, 15, 16^b^, 18, 19
	DNA dependent RNA polymerase	rpoA, B, C1^b^, C2
	rRNA genes	rrn4.5^c^, 5^c^, 16^c^, 23^c^
	tRNA genes	trnA-UGC^b,c^, C-GCA, D-GUC, E-UUC, F-GAA, G-UCC, G-GCC^b^, H-GUG, I-CAU^c^, I-GAU^b,c^, K-UUU^b^, L-UAG, L-CAA^c^, L-UAA^b^, M-CAU, fM-CAU, N-GUU^c^, P-UGC, Q-UUG, R-ACG^c^, R-UCU, S-GGA, S-GCU, S-UGA, T-GGU, T-UGU, V-UAC^b^, V-GAC^c^, W-CCA, Y-GUA
Photosynthesis	Photosystem I	psaA, B, C, I, J, ycf3^a^, ycf4
	Photosystem II	psbA, B, C, D, E, F, H, I, J, K, L, M, N, T, Z
	NADH oxidoreductase	ndhA^b^, B^b,c^, C, D, E, F, G, H, I, J, K
	Cytochrome b6/f complex	petA, B^b^, D^b^, G, L, N
	ATP synthase	atpA, B, E, F^b^, H, I
	Rubisco	rbcL
Other gene	Translational initiation factor	infA
	Maturase	matK
	Protease	clpP^a^
	Envelop membrane protein	cemA
	Subunit Acetyl-CoA-carboxylase	accD
	c-type cytochrom synthesis gene	ccsA
Unknown gene	Conserved Open Reading Frames	ycf1, 2^c^,15^c^, orf42

^a^Genes containing two introns;

^b^Genes containing a single intron;

^C^Two gene copies in the IRs;

^d^Genes split into two independent transcription units.

**Figure 2:**
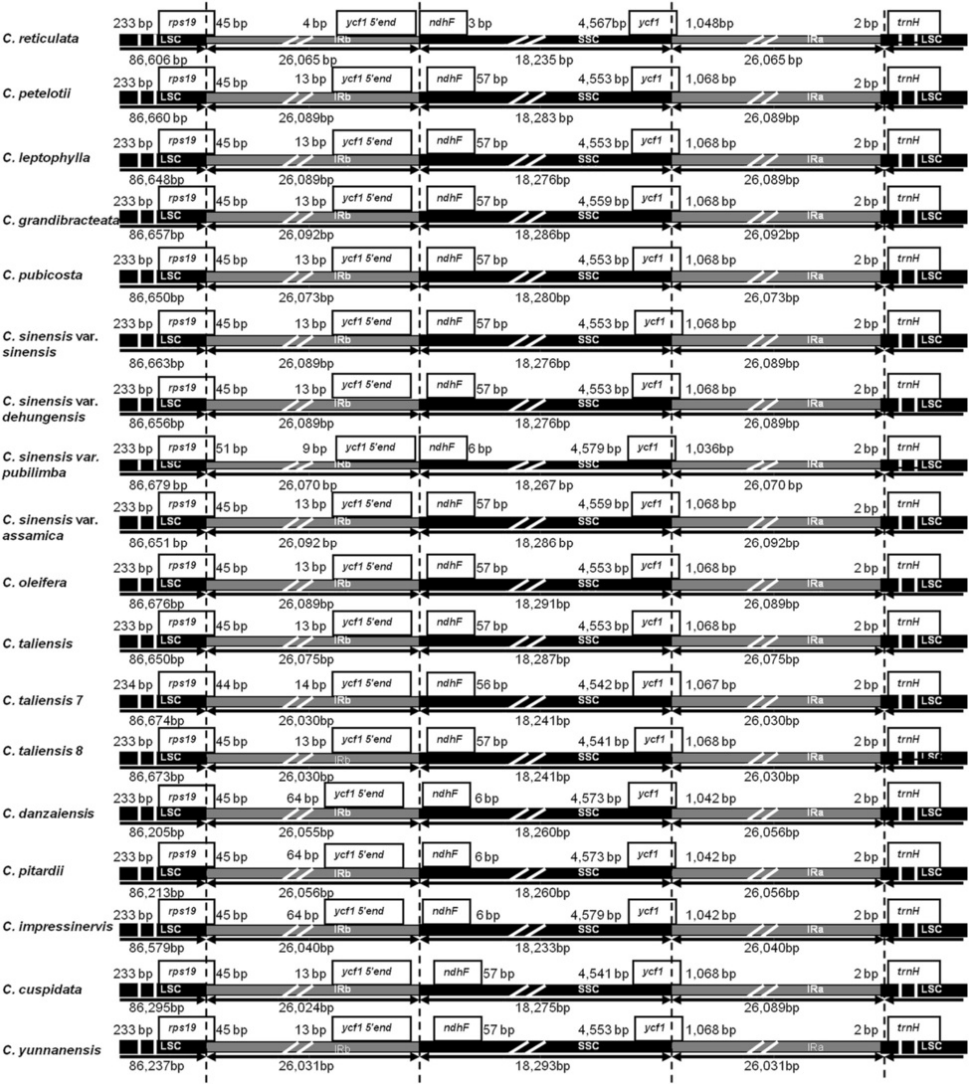
**The comparison of the LSC, IR and SSC border regions among the eighteen *Camellia *** **chloroplast genomes.**

Although genome size and overall genomic structure including gene number and gene order are well conserved, IR expansion/contraction is common in plant cp genomes. In grasses, for example, the termini of two genes, *ndhH* and *ndhF*, were reported to have repeatedly migrated into and out of the adjacent IRs [22]. Whole *rps19* was located within the LSC region in the most *Gossypium* cp genomes but failed to find in cp genome of *G. raimondii* D5 [21]. Kim et al. [23] considered that the length of angiosperm cp genomes is variable primarily due to the expansion and contraction of the inverted repeat IR region and the single-copy boundary regions. The IR/SC boundary regions of the 18 complete *Camellia* cp genomes were compared, showing slight differences in junction positions (Figure 2). The junction positions were conserved across 12 *Camellia* cp genomes and were variable in cp genomes of *C. taliensis 7, C. reticulata, C. sinensis* var. *pubilimba, C. danzaiensis*, *C. pitardii* and C*. impressinervis.* For example, the distances from *ndhF* and *ycf1 5′ end* to the junction of IRb/SSC were 3 and 4 bp, respectively in *C. reticulata*, and 6 and 9 bp, respectively, in *C. sinensis* var. *pubilimba* due to the deletion. The distances were 6 and 64 bp, respectively, in *C. danzaiensis*, *C. pitardii* and C*. impressinervis*, which is different from the distances of 57 and 13 bp, respectively, in other 12 *Camellia* cp genomes. The gene *ycf1* extended into the IRa region with 1,068 bp in the 12 cp genomes, with 1,048 bp in *C. reticulata*, with 1,067 bp *in C. taliensis 7*, with 1,036 bp in *C. sinensis* var. *pubilimba*, and with 1,042 bp in *C. danzaiensis*, *C. pitardii* and C*. impressinervis* cp genomes.

**Figure 3:**
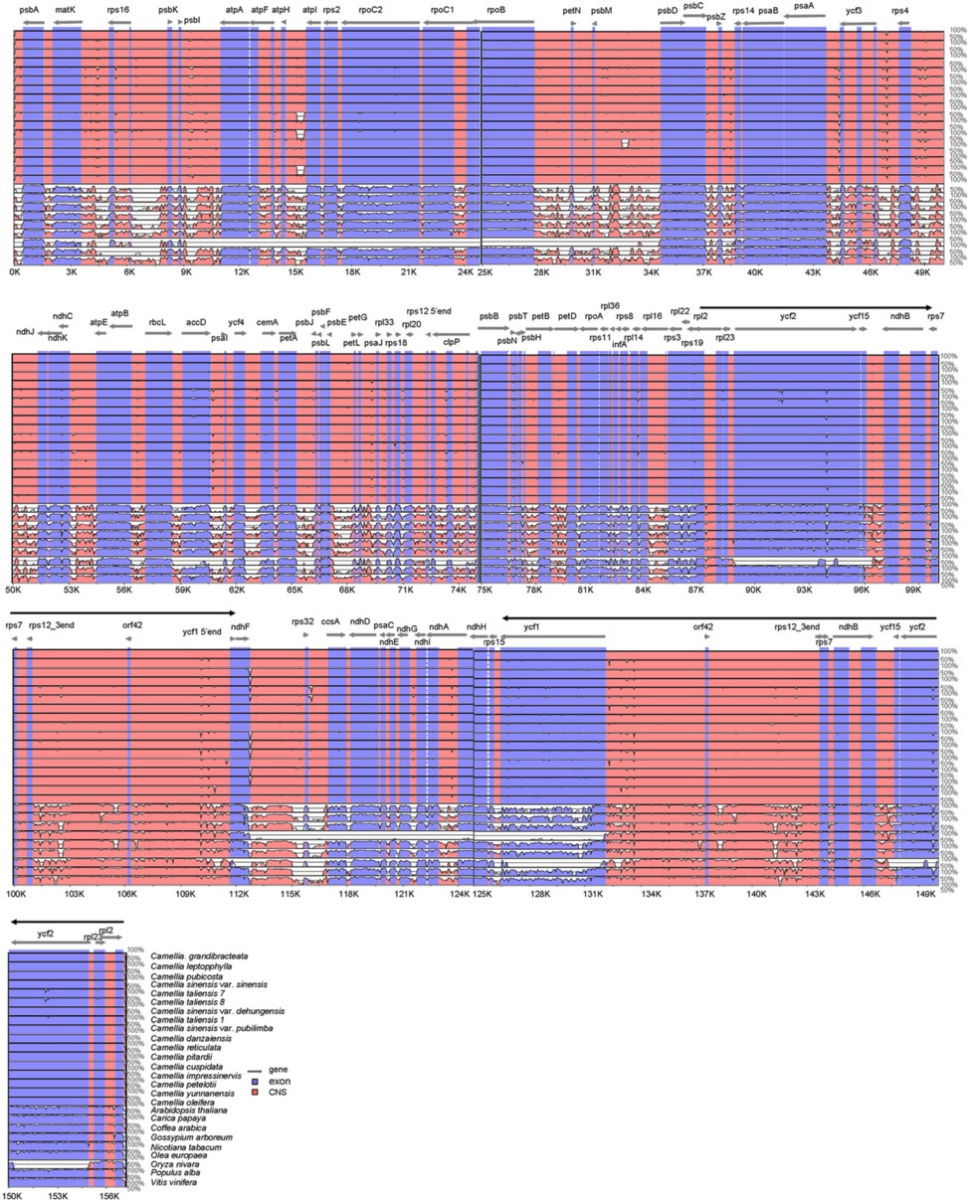
**Visualization alignment of chloroplast genome sequences.** VISTA-based identity plots showing sequence identity between the eighteen sequenced *Camellia* chloroplast genomes and nine other representative flowering plants, with *Camellia sinensis* var. *assamica* as a reference. Thick black lines show the inverted repeats (IRs) in the chloroplast genomes. Genome regions are color-coded as protein coding, rRNA coding, tRNA coding or conserved noncoding sequences (CNS).

To investigate levels of genome divergence, multiple alignments of 18 *Camellia* cp genome sequences and nine representative plants with fully sequenced cp genomes were performed (Figure 3 and Additional file 2: Table S2). With *C. sinensis* var. *assamica* as a reference, we plotted sequence identity using VISTA [24]. The results revealed high sequence similarity across the 18 *Camellia* cp genomes, suggesting that *Camellia* cp genomes are rather conserved. However, the marked differences were observed between *Camellia* cp genomes and other plants, including *Coffea arabica.* As expected, the IRs are more conserved than single-copy regions, and coding regions are more conserved than noncoding regions. The most divergent coding regions were *matK*, *rpoC2*, *accD*, *rps19, ycf2* and *ycf1* (Figure 3). Considering high conservation of *Camellia* cp genomes, as above described, we only included *C. sinensis* var. *assamica* (ASSA, sect. *Thea*), *C. oleifera* (OLEI, sect. *Paracamellia*), *C. reticulata* (RETI, sect. *Camellia*), *C. petelotii* (PETE, sect. *Archecamellia*) and *C. pubicosta* (PUBI, sect. *Corallina*) that represent a wide phylogenetic diversity to compare and characterize their cp genomic structural variations (Additional file 3: Figure S1).

### Repetitive sequences

**Figure 4:**
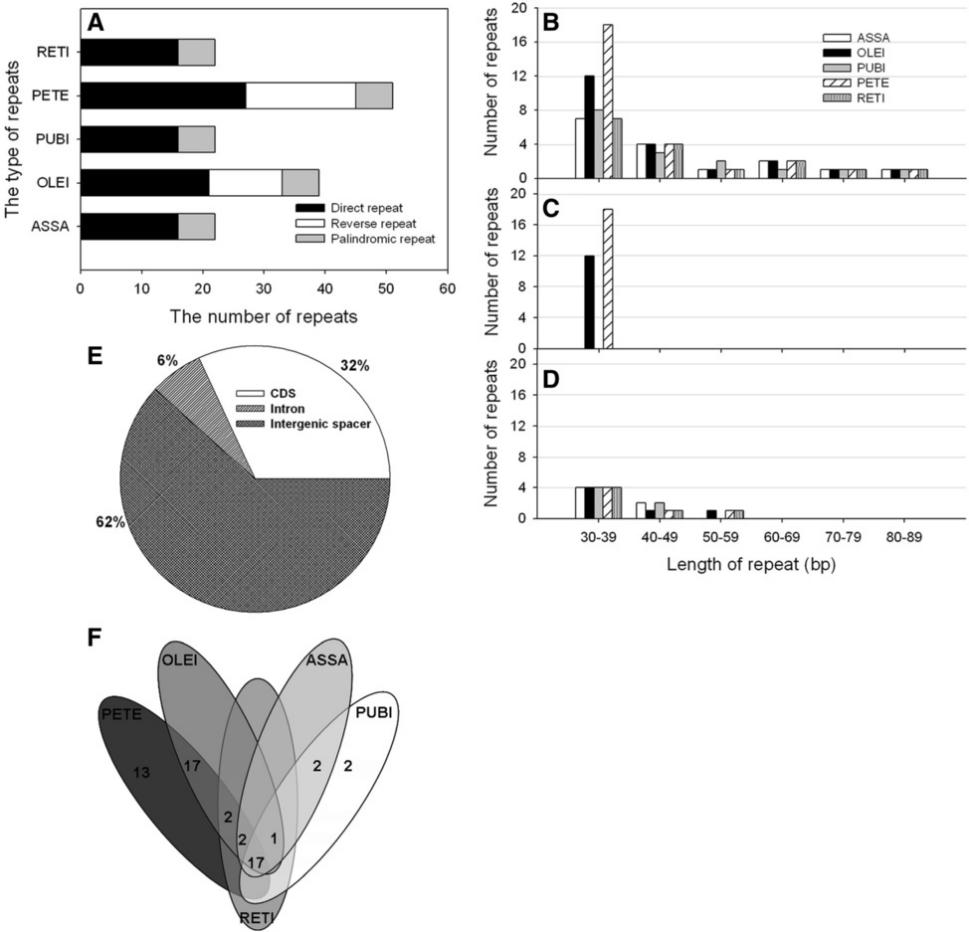
**Analyses of repeated sequences in the five *Camellia *chloroplast genomes. A** Number of the three repeat types; **B** Frequency of the direct repeats by length; **C** Frequency of the reverse repeats by length; **D** Frequency of palindromic repeats by length; **E** Location of repeats; **F** Summary of shared repeats among the five *Camellia* chloroplast genomes. IGS, intergenic spacer.

Repeated sequences are generally considered to be uncommon in cp genomes with the notable exception of a large IR present in most land plants [25]. In order to avoid redundancy, repeat sequences analysis in the five *Camellia* cp genomes mentioned above was carried out with a single IR region. A total of 156 repeats were detected in these cp genomes using REPuter [26], including direct, reverse and palindromic repeats (Additional file 4: Table S3). Number and distribution of repeats are rather conserved between cp genomes of ASSA, PUBI and RETI, excluding reverse repeats. Nevertheless, OLEI and PETE cp genomes included three repeat types, that is, direct, reverse and palindromic repeats (Figure 4). Among them, direct repeats are the most common, accounting for 62% of the total repeats, followed with palindromic repeat (19%) and reverse repeat (19%) (Figure 4A). The lengths of repeats in these five *Camellia* cp genomes were much shorter, ranging from 30 to 82 bp (Figure 4B, C and D), whereas much longer repeats, such as 132-bp and 287-bp repeats were found in the Poaceae and Fabaceae [27-29]. Palindromic and reverse repeats occurred in a narrower size, ranging from 30–50 bp and 30–35 bp, respectively. In this study, although a minority of repeats was found in intron (6%), the majority were located in IGS (62%) and coding sequence (CDS) regions (32%) (Figure 4E). Then, we investigated the repeats shared among the five *Camellia* cp genomes. Here, we defined repeats that had identical lengths and located in homologous regions as shared repeats. Under such criteria there were 17 repeats shared by the five *Camellia* cp genomes and three repeats were presented jointly in four cp genomes. PETE had the most unique repeats (13), while ASSA, OLEI and RETI showed no unique repeats (Figure 4F). Previous work suggested that repeat sequences have played an important role in genomic rearrangement and sequence variation in cp genomes through illegitimate recombination and slipped-strand mispairing [30-32]. The existence of these repeats implies that the region is a potential hotspot for genomic reconfiguration [33]. Our results also showed that divergent regions of cp genomes were associated with various repeat sequences such as intergenic *atpF/atpH*. These repeats may further serve as genetic markers for phylogenetic and population genetic studies.

### SSR polymorphisms

**Figure 5:**
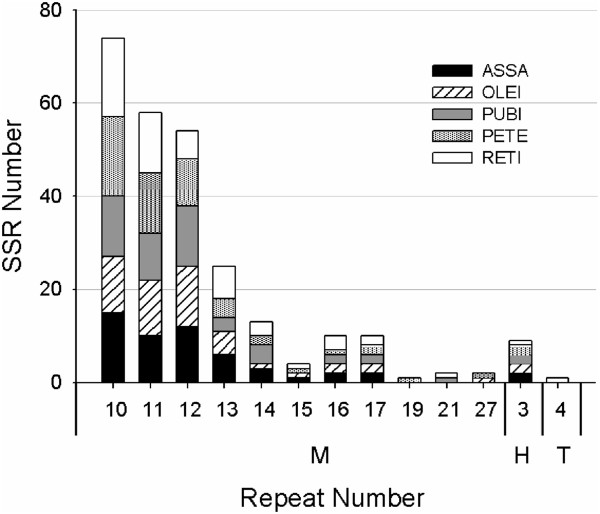
**The distribution of simple sequence repeats (SSRs) in the five *Camellia *chloroplast genomes.** M: Mononucleotide; T: Tetranucleotide; H: Hexanucleotide.

SSRs usually have a higher mutation rate compared to other neutral DNA regions due to slipped DNA strands. They thus are often used as genetic markers, providing useful information concerning plant population genetics and ecological and evolutionary studies due to their non-recombinant, haploid and uniparentally inherited nature [34,35]. In total, 53, 51, 50, 55 and 55 SSRs were found in the cp genomes of ASSA, OLEI, PUBI, PETE and RETI, respectively (Figure 5, Table 4 and Additional file 5: Table S4). Mononucleotide (A/T) and hexanucleotide (AAAAAG/CTTTTT) repeats were detected in the five *Camellia* cp genomes. One tetranucleotide (AGGG/CCCT) repeat was only found in RETI cp genome, and no dinucleotide and trinculeotide repeats were observed. The repeat unit A/T was found to be the most abundant with particular repeat numbers of 10, 11 and 12 (Figure 5). The finding is consistent with a previous observation that cp SSRs were dominated by A or T mononucleotide repeats [36]. Mononucleotide and hexanucleotide repeats were composed of A or T at a higher level, which reflects a biased base composition with an overall A-T richness in the cp genomes [28,37]. Within the five *Camellia* cp genomes, SSR loci mainly located in IGS, following by CDS and introns. There were A/T (12) SSRs located in CDS-IGS (*psbI, psbI/trnS-GCU*) expect for PETE. No SSRs were found in the tRNAs and rRNAs. We observed that 11 SSRs located in seven protein-coding genes [*ycf1* (×5)*, ccsA, rpoB, atpB, rpoA, rpoC2, ndhK*] of the five *Camellia* cp genomes. Jakobsson et al. [38] indicated that cp SSRs located in the non-coding regions of the cp genome commonly show intraspecific variation in repeat number. Most of those SSRs loci were located in LSC region, followed by IR and SSC regions. We found that 11 SSR loci were located in IRs of the five *Camellia* cp genomes. This observation is surprising because concerted evolution, as suggested earlier, should lead to exact sequence duplication in IRa compared with IRb, and therefore both IRs should contain the same number of nucleotide repeats. This may be explained by the incomplete repeat of *ycf1* in IRb that led to the five SSRs located in IRb and inexactly identical SSRs between IRa and IRb. Length variations in SSRs have served as useful markers for identifying crop varieties and performing population genetic studies [39,40]. cp SSRs characterized in this study could undoubtedly provide an assay for detecting polymorphisms at the population-level and comparing more distantly phylogenetic relationships at the genus level or above.

**Table 4:** **Simple sequence repeats (SSRs) in the five representative *Camellia *** **chloroplast genomes**

**Genomes**	**Repeat units**	**Number**	**Location**				**Region**		
			**Intron**	**IGS**	**CDS-IGS**	**CDS**	**LSC**	**SSC**	**IR**
ASSA									
	A/T	51	5	34	1	11	39	3	9
	AAAAAG/CTTTTT	2		2					2
OLEI									
	A/T	49	6	31	1	11	36	4	9
	AAAAAG/CTTTTT	2		2					2
PUBI									
	A/T	48	5	31	1	11	35	4	9
	AAAAAG/CTTTTT	2		2					2
PETE									
	A/T	53	7	35	0	11	41	3	9
	AAAAAG/CTTTTT	2		2					2
RETI									
	A/T	53	7	34	1	11	40	4	9
	AGGG/CCCT	1		1					1
	AAAAAG/CTTTTT	1		1					1

ASSA, *C. sinensis* var. *assamica*; OLEI, *C. oleifera*; PUBI, *C. pubicosta*; PETE, *C. petelotii*; RETI, *C. reticulata*.

### Substitution and indel variation

Global alignment of the five *Camellia* cp genomes revealed that total substitutions varied from 82 (ASSA *vs.* PUBI) to 265 (ASSA *vs.* OLEI) (Table 5). The base substitution types between C and G were fewer than other types, in agreement with a previous study [40]. A comparison of indels among these cp genomes (Table 5) showed that the number of indels ranged from 28 (ASSA *vs.* PUBI) to 72 (PUBI *vs.* RETI). These indel events were mainly attributed to the repetition of an adjacent sequence, probably caused by slipped-strand mis-pairing in DNA replication [41]. Indels are thought to be a major driving force in sequence evolution [42,43]. We observed that there were the fewest substitutions and indels between ASSA and PUBI. The ratios of nucleotide substitution events to indel events (S/I) for different pairwise comparisons showed that, among the five *Camellia* cp genomes, the S/I ratio varied from 2.67 (PETE *vs.* RETI) to 5.00 (ASSA *vs.* OLEI) (Table 5). Given the S/I ratio increased with divergence times between genomes [44], ASSA was inferred to be close to PUBI (S/I = 2.93), suggesting that PUBI may be classified into sect. *Thea*. The likely explanation is that the S/I ratio increased with the increase of divergence times that may arise from systematic underestimation of indels in more distantly related species [44].

**Table 5:** **The numbers and ratios of nucleotide substitutions and indels in the five *Camellia *** **chloroplast genomes**

**Genomes**	**ASSA**	**OLEI**	**PUBI**	**PETE**	**RETI**
**ASSA**	/	53	28	57	69
**OLEI**	265 (5.00)	/	61	45	65
**PUBI**	82 (2.93)	252 (4.13)	/	65	72
**PETE**	217 (3.81)	204 (4.53)	193 (2.97)	/	64
**RETI**	242 (3.51)	224 (3.45)	221 (3.07)	171 (2.67)	/

The upper triangle shows the number of indels, while the lower triangle indicates the total nucleotide substitutions. The ratios of nucleotide substitutions to indels (S/I) are given in brackets.

**Figure 6:**
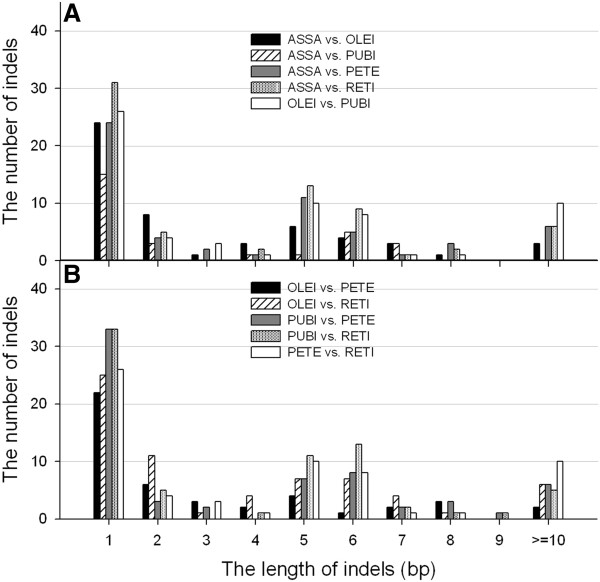
**The distribution of indel types in the five *Camellia *chloroplast genomes.** The pairwise comparisons were performed to identify indels among the five *Camellia* chloroplast genomes. **A** includes ASSA *vs*. OLEI/PUBI/PETE/RETI and OLEI *vs*. PUBI. **B** includes OLEI *vs*. PETE/RETI, PUBI *vs*. PETE/RETI and PETE *vs*. RETI.

The number of short indels (1–10 bp) accounted for >90% of total indels (Figure 6). As expected, single-nucleotide (1-bp) indels were the most common, accounting for approximately 38% (OLEI *vs.* RETI) to 54% (ASSA *vs.* PUBI) of all indels. Xu et al. [21] concluded that 1–3 bp indels were mainly attributed to the SSR polymorphisms. Yamane et al. [45] and McCluskey et al. [46] observed that the number of indels decreased rapidly with the increase of indel lengths. However, we observed that the 5-6-bp indels were the almost second most abundant of all characterized indels, except for OLEI, rather than 2-bp indels, and the number of 5-6-bp indels was apparently more than that of 3-4-bp indels. It is likely that such 5-6-bp indels were caused by adjacent 5-6-bp motif duplications or losses, making it the second most common type [21].

### Molecular marker identification

**Figure 7:**
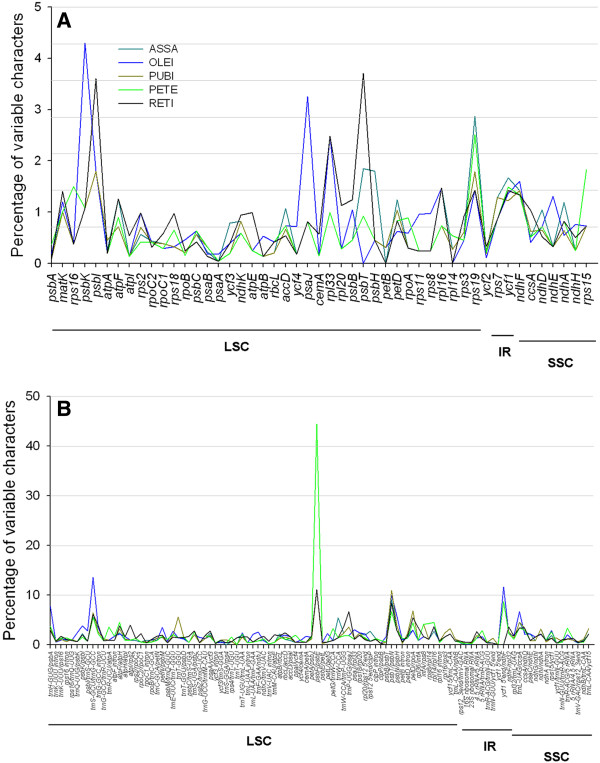
**Percentages of variable characters in homologous regions across the five *Camellia *chloroplast genomes. A** Coding regions; **B** Non-coding regions.

Molecular evolutionary rates are often associated with life history in flowering plants [47]. The *Camellia* species with rather long-generation times may have evolved slowly. Considering that a low rate of molecular evolution could complicate the phylogenetic analysis of *Camellia*, the identification of rapidly evolving cp genomic regions is critical through comparative genomic analysis. For purposes of the subsequent evolutionary and phylogenetic analyses, whole cp genome annotation and sequence comparisons showed that the number and distribution patterns of variable characters in coding and non-coding regions were fairly different among the five *Camellia* cp genomes (Figure 7). Among them, the proportions of variability in non-coding regions ranged from 0 to 44.4% with a mean value of 1.79%, which were twice as much as in the coding regions (0.72% on average). Fewer mutations were observed within IR regions, including coding and non-coding regions, than LSC and SSC regions. For coding regions, the remarkably high proportions of variability of *rps19* were observed in all five *Camellia* cp genomes. The high variability of gene *rps19* might result from their extension into IR region where intrachromosomal recombinations frequently occurred to ensure the stability and consistency of IRs [25]. The proportion of variability in *Camellia* was lower than that in grasses [28]. We thus chose the 15 most variable non-coding regions that may serve as candidate markers for phylogenetic reconstruction (Additional file 6: Table S5), which were identified with variations that exceed 1.5% in the five *Camellia* cp genomes. They were *trnH-GUG/psbA, psbK/psbI, trnS-GCU/trnG-GCC, trnG-GCC* intron, *atpF/atpH, trnE-UUC/trnT-GGU, trnS-UGA/psbZ, psaA/ycf3, trnP-UGG/psaJ, trnT-UGU/trnL-UAA, rps18/rpl20, petD/rpoA, ycf15/trnL-CAA, ndhF/rpl32* and *ccsA/ndhD*. Two of them were located in SSC region (*ndhF/rpl32,* and *ccsA/ndhD*) and the *ycf15/trnL-CAA* was located in IR regions. However, the determination whether these 15 regions could be applied to phylogenetic analyses in *Camellia* requires further studies.

### Structural constraints on evolutionary divergence

**Figure 8:**
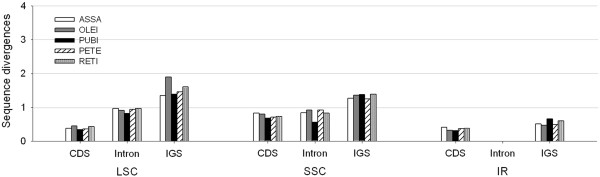
**Levels of evolutionary divergences among different regions of the five *Camellia *** **chloroplast genomes.**

Previous studies suggested that evolutionary differences in the cp genomes are dependent on the sequence and gene functions [37] and related to the structural constraints [37,48]. An alignment was performed among the CDS, introns, and IGS regions, along with positional information of the five *Camellia* cp genomes. The sequence divergence ratios among the three regions (CDS:intron:IGS) were 1:1.1:1.9 (ASSA: four other species), 1:1.2:2.4 (OLEI: four other species), 1:1:2.6 (PUBI: four other species) , 1:1.3:2.2 (PETE: four other species) and 1:1.2:2.3 (RETI: four other species), respectively (Figure 8). The result clearly suggests that the intron sequences have evolved faster than the CDS but slower than the IGS sequences. The rapid evolution of intron sequence was attributed to sequence divergence ratios in LSC and SSC (Additional file 7: Figure S2). This finding is also supported by a previous statement that the nucleotide substitution rates in the IGS sequences and introns are higher than the CDS [49]. The sequence alignment data sets were further partitioned into IR, LSC and SSC regions, and the sequence divergence ratios among the three regions (IR:LSC:SSC) were found to be 1:2.9:3.2 (ASSA: four other species), 1:4.1:3.9 (OLEI: four other species), 1:2.6:2.7 (PUBI: four other species) , 1:3.2:3.3 (PETE: four other species) and 1:3.1:3.0 (RETI: four other species), respectively (Figure 8). Such comparisons apparently indicate that the IR regions may have evolved much more slowly than the LSC and SSC regions, and levels of evolutionary divergence of introns in IRs were much lower than those of introns in LSCs and SSCs (Additional file 7: Figure S2). The frequent intrachromosomal recombination events between these two identical IR regions of the cp genome provide selective constraints on both sequence homogeneity and structural stability [25,50]. This could be used to explain why the IR regions exhibit slow nucleotide substitution rates in comparison with the SSC and LSC regions in this study. Our results thus confirm that positional effects are stronger constraints for sequence evolution than the functional groups of chloroplast genes, in good agreement with the previous observation [37].

### Phylogenomic analyses

**Figure 9:**
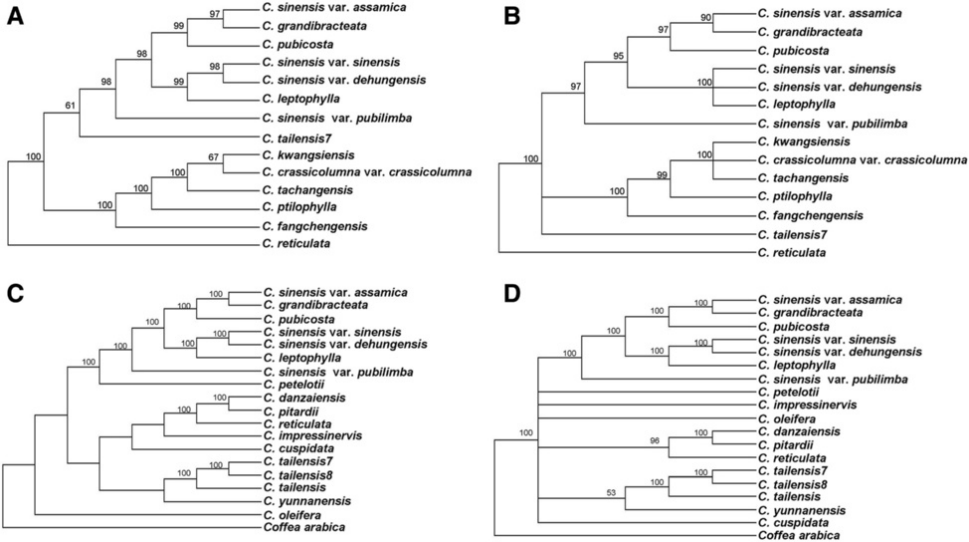
**Phylogenetic relationships of the thirteen species of section *Thea *and the eighteen species of *Camellia *constructed by maximum likelihood (A, C) and maximum parsimony (B, D) with *C. reticulata *and *Coffea arabica *as outgroup.** The A and C ML trees have a -InL = 124830.0859 and -InL = 290325.4563. The B MP tree has a length of 1,129 with a consistency index of 0.890 and a retention index of 0.766. The D MP tree has a length of 14,892 with a consistency index of 0.983 and a retention index of 0.796. Numbers above node are bootstrap support values (>50%).

The phylogenetic studies based on the cp genome sequences are addressed successfully for the phylogenetic issues of angiosperm [12,28,51]. The species in the sect. *Thea* have long been considered to be a complex and taxonomically difficult group because of their alike morphological characteristics. Chang et al. [52] classified the sect. *Thea* into a total of 4 series, which comprised 42 species and four varieties. However, Min et al. [2] proposed to taxonomically classify this section into 12 species and 6 varieties. In particular, there is a controversy on the taxonomy of *C. pubicosta* native to Laos. This species was classified into sect. *Thea* by Chang et al. [52], while Min et al. [53] insisted to classify into sect. *Corallina* considering that some characters of *C. pubicosta* are different with that of other members in sect. *Thea*. Using *C. reticulata* that belongs to sect. *Camellia* as outgroup, our phylogenetic analysis of orthologous sequences from the sampled species in this study and recently sequenced species (*C. taliensis 7*) of sect. *Thea* showed that the ML tree was mostly consistent with MP tree with high bootstrap supports, except for the position of *C. taliensis* (Figure 9). It is notable that *C. pubicosta* was sister to *C. sinensis* var. *assamica* and *C. grandibracteata* with BS = 100% (Figure 9A and B), supporting Chang’s taxonomical treatment that *C. pubicosta* was classified into sect. *Thea*. Previous phylogenetic analysis using RAPDs reported that the species of sect. *Thea* could be divided into two groups, consistent with the number of locule ovary, that is, 5-locule ovary group and 3-locule ovary group [5,54]. As the first well-supported phylogenomic analyses of sect. *Thea*, however, our results evidently demonstrated that phylogenetic relationships and molecular evolution of the species in sect. *Thea* did not well follow the number of locule ovary. For example, *C. fangchengensis, C. ptilophylla*, *C. tachangensis, C. kwangsiensis* and *C. crassicolumna* var. *crassicolumna* were well supported as monophyletic. However, *C. fangchengensis* and *C. ptilophylla* belonged to Ser. *Sinenses* with 3 ovaries, while *C. tachangensis, C. kwangsiensis* and *C. crassicolumna* var. *crassicolumna* were members of Ser. *Quinquelocularis* and Ser. *pentastylae* with 5 ovaries. Our results thus indicated that taxonomical value of the number of ovary may be reconsidered to classify the *Camellia* species.

The phylogenetic analyses were performed based on the entire cp genome sequences from 18 *Camellia* cp genomes (Figure 9C and D), showing that the species of sect.*Thea* formed a monophyletic clade, except for the three individuals of *C. taliensis*, which is close to *C. yunnanensis*. This result indicated that *C. taliensis* may not be the ancestors of *C. sinensis* var. *assamica*[16] and there might be hybridization between *C. taliensis* and *C. yunnanensis* due to chimeric habitats. We observed that *C.danzaiensis*, *C.pitardii* and *C.reticulata* formed a monophyletic clade with strong bootstrap support, which might suggest that *C. danzaiensis* belongs to Subgen. *Camellia*, rather than Subgen. *Thea*. The different positions of *C. impressinervis* and *C. cuspidate* in ML and MP tree made more samples to resolve their phylogenetic relationship is essential. Further genomic and taxon sampling and more complete cp genomes of *Camellia* are deserved in further studies as phylogenomic analysis tends to suffer from the poor sampling [55].

**Figure 10:**
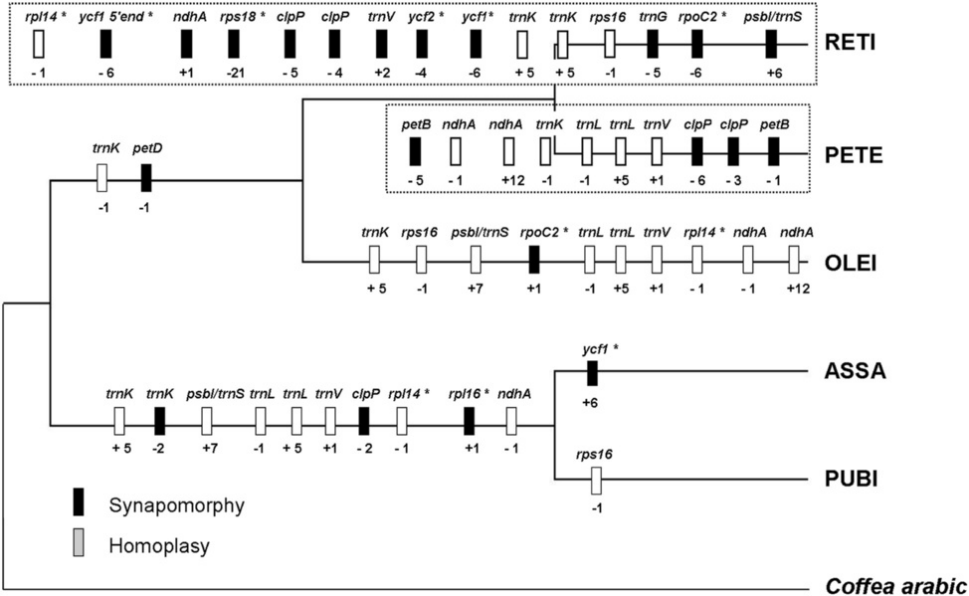
**Distribution of indels within introns and coding sequences of the five *Camellia *chloroplast genomes.** The phylogenetic tree was a subtree of Figure 10 using *C. arabic* as outgroup. The insertions are indicated as ‘+’ and deletions are marked as ‘-’ on the branch. The genes are designated as ‘*’. Synapomorphy and homoplasy are shown by black and gray bars, respectively.

Indels not only play an important role in elucidating genome evolution [20,42], but also have potential value in constructing phylogenies [56,57]. A total of 63 putative informative indels were identified by pairwise comparisons, and then mapped to the cp genome-based phylogenetic tree using *C. arabic* as outgroup (Figure 10). Of these, 46, 13 and 4 indels were located in introns, CDS and CDS-IGS, respectively. Among these five branches, the branch resulting in RETI contained the most number of indels (17 of 63). We observed the most number of indels that were shared between ASSA (10/11) and PUBI (10/11), suggesting their short divergence times and close relationships. Of all indels, 26 were able to be mapped to phylogenetic tree with high bootstrap supports and thus are indicative of synapomorphies. The remaining 37 indels may be homoplasies possibly associated with parallel mutations or back mutations during evolutionary history, which somehow had negative effects on the reconstruction of phylogenetic tree. Such indels should be carefully used especially when a few number of DNA fragments were applied for phylogenetic studies.

## Conclusions

We reported eight complete and five draft cp genomes in the genus *Camellia* using Illumina sequencing technology via a combination of *de novo* and reference-guided assembly. These cp genomes were found highly conserved each other. We investigated the variation of repeat sequences, SSRs, indels and substitutions among the five complete *Camellia* cp genomes, representing a wide phylogenetic diversity in the genus *Camellia*. The fifteen rapidly evolving regions were identified across these cp genomes that could serve as potential molecular markers for further phylogenetic studies. This study is undoubtedly the first successful attempt to provide well-supported evolutionary relationships of sect. *Thea* based on phylogenomic analyses. The obtained cp genomes may facilitate the development of biotechnological applications for these economically important woody plants, and offer useful genetic information for purposes of phylogenetics, taxonomy and species identification in the genus *Camellia*.

## Methods

### Plant materials

Leaf materials of the *Camellia* plants used in this study were collected from Kunming Institute of Botany (Chinese Academy of Sciences), Tea Research Institute (Yunnan Academy of Agricultural Sciences) and International *Camellia* Species Garden (Jinhua, Zhejiang Province, China) in May 2011 (Table 1). The collected plant materials were classified by Min’s taxonomic treatment [2] (Table 1). *C. gymnogyna* and *C. costata* of sect. *Thea* were unavailable and thus were absent in this study.

### DNA sequencing and genome assembly

Approximately 20 g of fresh leaves from each species were harvested for cpDNA isolation using an improved extraction method that includes high ionic strength buffer at low pH (3.8) [58]. After DNA isolation, 5 μg of purified DNA was fragmented by nebulization with compressed nitrogen gas, and constructed short-insert (300 bp) libraries following the manufacturer’s protocol (Illumina). DNA from the different species was indexed by tags and pooled together in one lane of Illumina’s Genome Analyzer for sequencing (2 × 100 bp) at Germplasm Bank of Wild Species in Southwest China, Kunming Institution of Botany, Chinese Academy of Sciences. Raw reads were first filtered to obtain the high-quality clean data by removing adaptor sequences and low-quality reads with Q-value ≤ 20. Then, those reads mixed non-cp DNA from the nucleus and mitochondria were isolated based on the known cp genome sequences. Then, the following three steps were used to assemble cp genomes [19]. First, the filtered reads were assembled into contigs using SOAPdenovo [59]. Second, contigs were aligned to the reference genome of *C. sinensis* var. *assamica* (Genbank ID: JQ975030) using BLAST, and aligned contigs (≥90% similarity and query coverage) were ordered according to the reference genome. Third, raw reads were again mapped to the assembled draft cp genomes that were then visualized by Geneious (version 5.1) [60], and the majority of gaps were filled through local assembly.

Based on the reference genome of *C. sinensis* var. *assamcia*, we designed four primer pairs for the verification of the four junctions between the single-copy segments and IRs (as given in Additional file 1: Table S1), respectively. PCR products were then sequenced following standard Sanger protocols on ABI 3730 ×1 instruments. Sanger sequences and assembled genomes were aligned using Geneious assembly software to determine if there were any differences.

### Genome annotation, alignment and visualization

The chloroplast genes were annotated using an online DOGMA tool [61], using default parameters to predict protein-coding genes, transfer RNA (tRNA) genes, and ribosome RNA (rRNA) genes. Start and stop codons of protein-coding genes were searched and determined by BLASTX against the NCBI protein database, with *C. sinensis* var. *assamica* as a guide. Genome maps were drawn with OGDraw (version 1.2) [62]. Multiple alignments were made using MAFFT version 5 [63] and adjusted manually where necessary. Full alignments with annotations were visualized using the VISTA viewer [24].

### Characterization of repeat sequences and SSRs

REPuter [26] was used to identify and locate the repeat sequences, including direct, reverse and palindromic repeats within cp genome. For repeat identification, the following constraints were set to REPuter: (i) minimum repeat size of 30 bp, and (ii) 90% or greater sequence identity, based on Hamming distance of 3.

SSRs were predicted using MISA [64] with the parameters set to ten repeat units ≥10 for mononucleotide SSRs, six repeat units ≥6 for dinucleotide, five repeat units ≥5 for trinucleotide, four repeat units ≥4 for tetranucleotide, and three repeat units ≥3 for pentanucleotide and hexanucleotide SSRs.

### Identification of molecular markers

To identify the divergent regions for phylogenetic analyses, all the regions, including CDS, introns and IGS from the *Camellia* cp genomes, were sequentially extracted. For each species, homologous regions of cp genomes were aligned using MAFFT version 5 and manual adjustments were made where necessary. Subsequently, the percentage of variable characters for each region was obtained. The proportion of mutational events (or variation%) was calculated by following the modified version of the formula used in Gielly and Taberlet [65]. The proportion of mutation events = [(NS + ID)/L] × 100, where NS = the number of nucleotide substitutions, ID = the number of indels, L = the aligned sequence length.

### Phylogenetic analysis

The *Camellia* cp genome sequences were aligned using the program MAFFT version 5 [63] and adjusted manually where necessary. The ambiguously aligned loci (e.g., ‘N’) were excluded from the analyses. The unambiguously aligned DNA sequences were used for the reconstruction of phylogenetic trees. The phylogenetic analyses were performed based on the following two data sets: (1) the remaining sequences with lengths from 83,585 to 83,835 bp (including 78.1% coding and 21.9% non-coding regions) after the removal of the ‘N’s in incomplete cp genomes as well as the corresponding orthologous sequences in complete cp genomes from the alignment of the 13 *Camellia* cp genomes that belong to sect. *Thea* with *C. reticulata* as outgroup; (2) the eight complete cp DNA sequences sequenced obtained in this study, three *Camellia* cp genomes adopted from [18], and seven *Camellia* cp genomes retrieved from [9] with *C. arabica* as outgroup.

ML analyses were implemented in RAxML version 7.2.6 [66]. RAxML searches relied on the general time reversible (GTR) model of nucleotide substitution with the gamma model of rate heterogeneity. Non-parametric bootstrapping used 1,000 replicates as implemented in the “fast bootstrap” algorithm of RAxML. MP analyses were performed with PAUP*4.0b10. Heuristic tree searches were conducted with 1,000 random-taxon-addition replicates and tree bisection-reconnection (TBR) branch swapping, with “multrees” option in effect. Non-parametric bootstrap analysis was conducted under 1,000 replicates with TBR branch swapping.

## Availability of supporting data

These cp genomes sequenced in this study are available the GenBank database under the accession numbers (KJ806274-KJ806286). The alignments and phylogenetic trees supporting the results of this article are available in the TreeBASE repository, http://purl.org/phylo/treebase/phylows/study/TB2:S16027?x-accesscode=e9a8a916b74d332d14f1954ca00a51f6&format=html.

## Abbreviations

AFLPs: Amplified fragment length polymorphisms; ASSA: *C. sinensis* var. *assamica*; BS value: Bootstrap support values; CDS: Coding sequences; CNS: Conserved noncoding sequences; cp: Chloroplast; IR: Inverted repeat; ISSR: Inter-simple sequence repeat; ITS: Internal transcribed spacer; IGS: Intergenic spacer; LSC: Large single copy; ML: Maximum likelihood; MP: Maximum parsimony; OLEI: *C. oleifera*; PETE: *C. petelotii*; PUBI: *C. pubicosta*; RAPD: Random amplified polymorphic DNA; RETI: *C. reticulata*; rRNA: Ribosomal RNA; S/I: Nucleotide substitutions events to indels events; SSC: Small single copy; SSRs: Simple sequence repeats; TBR: Tree bisection-reconnection; tRNA: Transfer RNA.

## Competing interests

The authors declare that they have no competing interests.

## Authors’ contributions

Conceived and designed the experiments: LZG. Performed the experiments: HH and SYM. Analyzed the data: HH, CS and YL. Contributed reagents/materials/analysis tools: HH. Wrote the paper: LZG and HH. All authors read and approved the final manuscript.

## Supplementary Material

Additional file 1: Table S1 Primers used for junction verification. Click here for file

Additional file 2: Table S2 The list of accession numbers of the chloroplast genome sequences reported in this study. Click here for file

Additional file 3: Figure S1 Phylogenetic tree of the fourteen sections in the genus *Camellia*. The indicated phylogenetic relationships of the genus were constructed by using morphological data and adopted from Min et al. [2]. The arrowheads indicated that the species (right) was classified into the section (left). The two subgenera recognized in *Camellia* are given on the right side of the figure. Click here for file

Additional file 4: Table S3 Repeat sequences identified in the five *Camellia* chloroplast genomes. Click here for file

Additional file 5: Table S4 SSRs characterized in the five *Camellia* chloroplast genomes. Click here for file

Additional file 6: Table S5 The information of candidate markers with the variations more than 1.5%. Click here for file

Additional file 7: Figure S2 The variable characters of the seventeen intron regions of the five *Camellia* chloroplast genomes. Click here for file
