# Supplementary material for: Thirteen Camellia chloroplast genome sequences determined by high-throughput sequencing: genome structure and phylogenetic relationships
Source: BMC Evol Biol. 2014 Jul 7;14:151. doi: 10.1186/1471-2148-14-151 (PMC4105164; doi:10.1186/1471-2148-14-151)
Supplement: Additional file 1: Table S1 — Primers used for junction verification. [file 1471-2148-14-151-S1.docx]

**Table S1.** Primers used for junction verification.

| **Primer** | **Junction** | **Forward sequence** | **Reverse sequence** |
| --- | --- | --- | --- |
| P1 | LSC/IRa | 5' TTGTAGGTATAATGGTGGAT3' | 5' AAGCGTCCTGTAGTAAGAG3' |
| P2 | IRa/SSC | 5' TCCGAGTGAATGGAAAGG3' | 5'AAGGCATCAAATATGTAGGG3' |
| P3 | SSC/IRb | 5' ATAAACTGGGTGGAAACG3' | 5'AAACAGGAACAAGAGGGA3' |
| P4 | IRb/LSC | 5' AAGCGTTGGCTAGGTAAG3' | 5' TCAGGAAGGCGTTATTGT3' |
