# Supplementary material for: Thirteen Camellia chloroplast genome sequences determined by high-throughput sequencing: genome structure and phylogenetic relationships
Source: BMC Evol Biol. 2014 Jul 7;14:151. doi: 10.1186/1471-2148-14-151 (PMC4105164; doi:10.1186/1471-2148-14-151)
Supplement: Additional file 2: Table S2 — The list of accession numbers of the chloroplast genome sequences reported in this study. [file 1471-2148-14-151-S2.docx]

**Table S2.** The list of accession number of the chloroplast genome sequences used in this study

| **Classification** | **Family** | **Taxon** | **GenBank Accession Number** |
| --- | --- | --- | --- |
| Monocots |  |  |  |
|  | Poaceae | *Oryza nivara* | NC_005973 |
| Rosids |  |  |  |
|  | Vitaceae | *Vitis vinifera* | NC_007957 |
|  | Salicaceae | *Populus alba* | NC_008235 |
|  | Malvaceae | *Gossypium arboreum* | NC_016712 |
|  | Caricaceae | *Carica papaya* | NC_010323 |
|  | Brassicaceae | *Arabidopsis thaliana* | NC_000932 |
| Asterids |  |  |  |
|  | Theaceae | *Camellia sinensis* var. *assamica* | JQ975030 |
|  | Theaceae | *Camellia oleifera* | JQ975031 |
|  | Theaceae | *Camellia taliensis* | JQ975032 |
|  | Theaceae | *Camellia impressinervis* | KF156835 |
|  | Theaceae | *Camellia danzaiensis* | KF156834 |
|  | Theaceae | *Camellia pitardii* | KF156837 |
|  | Theaceae | *Camellia cuspidata* | KF156833 |
|  | Theaceae | *Camellia taliensis_7* | KF156836 |
|  | Theaceae | *Camellia taliensis_8* | KF156839 |
|  | Theaceae | *Camellia yunnanensis* | KF156838 |
|  | Theaceae | *Camellia sinensis* var. *dehungensis* |  |
|  | Theaceae | *Camellia grandibracteata* |  |
|  | Theaceae | *Camellia leptophylla* |  |
|  | Theaceae | *Camellia fangchengensis* |  |
|  | Theaceae | *Camellia kwangsiensis* |  |
|  | Theaceae | *Camellia crassicolumna* var. *crassicolumna* |  |
|  | Theaceae | *Camellia ptilophylla* |  |
|  | Theaceae | *Camellia tachangensis* |  |
|  | Theaceae | *Camellia sinensis* var. *sinensis* |  |
|  | Theaceae | *Camellia sinensis* var. *pubilimba* |  |
|  | Theaceae | *Camellia pubicosta* |  |
|  | Theaceae | *Camellia petelotii* |  |
|  | Theaceae | *Camellia reticulata* |  |
|  | Rubiaceae | *Coffea arabica* | NC_008535 |
|  | Oleaceae | *Olea europaea* | NC_013707 |
|  | Solanaceae | *Nicotiana tabacum* | NC_001879 |
