# Supplementary figures and images for: Thirteen Camellia chloroplast genome sequences determined by high-throughput sequencing: genome structure and phylogenetic relationships
Source: BMC Evol Biol. 2014 Jul 7;14:151. doi: 10.1186/1471-2148-14-151 (PMC4105164; doi:10.1186/1471-2148-14-151)

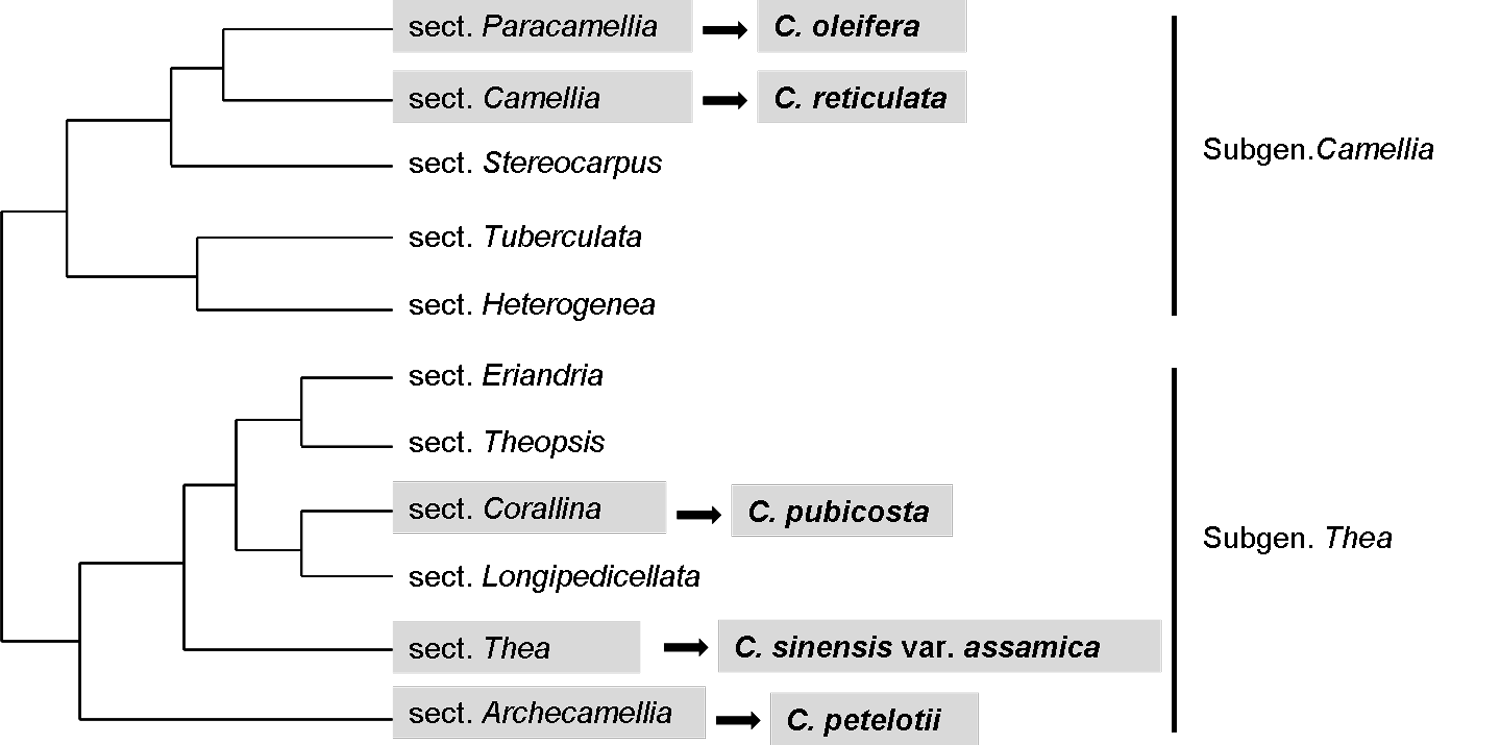

Supplement: Additional file 3: Figure S1 — Phylogenetic tree of the fourteen sections in the genus Camellia. The indicated phylogenetic relationships of the genus were constructed by using morphological data and adopted from Min et al. [2]. The arrowheads indicated that the species (right) was classified into the section (left). The two subgenera recognized in Camellia are given on the right side of the figure. [file 1471-2148-14-151-S3.tiff]

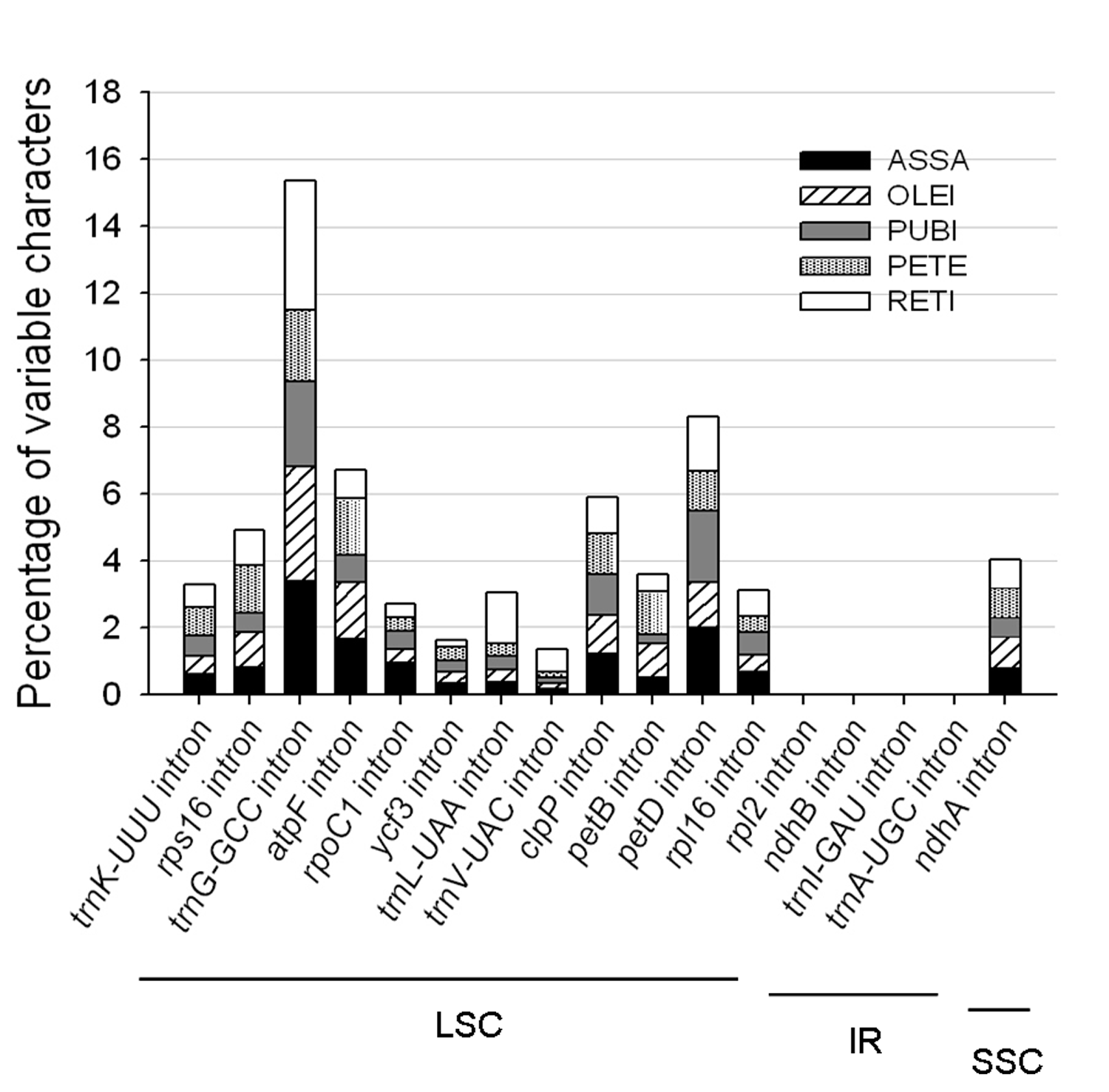

Supplement: Additional file 7: Figure S2 — The variable characters of the seventeen intron regions of the five Camellia chloroplast genomes. [file 1471-2148-14-151-S7.tiff]
