# Supplementary material for: Thirteen Camellia chloroplast genome sequences determined by high-throughput sequencing: genome structure and phylogenetic relationships
Source: BMC Evol Biol. 2014 Jul 7;14:151. doi: 10.1186/1471-2148-14-151 (PMC4105164; doi:10.1186/1471-2148-14-151)
Supplement: Additional file 4: Table S3 — Repeat sequences identified in the five Camellia chloroplast genomes. [file 1471-2148-14-151-S4.doc]

**Table S3.** A list of repeated sequences and their locations identified in the five *Camellia* chloroplast genomes.

| **Genomes** | **Repeat Type** | **Length**  **(bp)** | **Position A** | **Locus** | **Position B** | **Locus** | **Shared genomes** |
| --- | --- | --- | --- | --- | --- | --- | --- |
| **ASSA** | D | 82 | 93917 | *ycf2* | 93935 | *ycf2* | ALL |
|  | D | 70 | 93929 | *ycf2* | 93947 | *ycf2* | ALL |
|  | D | 60 | 93939 | *ycf2* | 93957 | *ycf2* | ALL |
|  | D | 60 | 93917 | *ycf2* | 93953 | *ycf2* | OLEI, PETE, RETI |
|  | D | 52 | 93929 | *ycf2* | 93965 | *ycf2* | ALL |
|  | P | 42 | 79208 | *petD intron* | 79208 | *petD intron* | PUBI |
|  | D | 42 | 101000 | *rps12_3end/trnV-GAC* | 122940 | *ndhA intron* | ALL |
|  | P | 45 | 76762 | *psbT/psbN* | 76762 | *psbT/psbN* | PUBI |
|  | D | 42 | 93939 | *ycf2* | 93975 | *ycf2* | ALL |
|  | D | 42 | 45592 | *ycf3 intron* | 122939 | *ndhA intron* | ALL |
|  | D | 42 | 93917 | *ycf2* | 93971 | *ycf2* | OLEI, PETE, RETI |
|  | D | 39 | 45595 | *ycf3 intron* | 101002 | *rps12_3end/trnV-GAC* | ALL |
|  | P | 30 | 9030 | *psbI/trnS-GCU, trnS-GCU* | 47309 | *trnS-GGA, trnS-GGA/rps4* | ALL |
|  | D | 38 | 60757 | *accD/psaI* | 60773 | *accD/psaI* | OLEI, PUBI, RETI |
|  | D | 34 | 93929 | *ycf2* | 93983 | *ycf2* | ALL |
|  | D | 35 | 40530 | *psaB* | 42754 | *psaA* | ALL |
|  | P | 30 | 14189 | *atpF/atpH* | 14189 | *atpF/atpH* | ALL |
|  | D | 32 | 9028 | *psbI/trnS-GCU, trnS-GCU* | 37373 | *psbC/trnS-UGA,* *trnS-UGA* | ALL |
|  | P | 31 | 38695 | *trnfM-CAU/* *rps14* | 38695 | *trnfM-CAU/rps14* | ALL |
|  | P | 30 | 37375 | *psbC/trnS-UGA,* *trnS-UGA* | 47309 | *trnS-GGA,* *trnS-GGA/rps4* | ALL |
|  | D | 30 | 45607 | *ycf3 intron* | 101014 | *rps12_3end/trnV-GAC* | ALL |
|  | D | 30 | 91481 | *ycf2* | 91523 | *ycf2* | ALL |
| **OLEI** | D | 82 | 93942 | *ycf2* | 93960 | *ycf2* | ALL |
|  | D | 70 | 93954 | *ycf2* | 93972 | *ycf2* | ALL |
|  | D | 60 | 93964 | *ycf2* | 93982 | *ycf2* | ALL |
|  | D | 60 | 93942 | *ycf2* | 93978 | *ycf2* | ASSA, PETE, RETI |
|  | P | 46 | 79233 | *petD intron* | 79233 | *petD intron* | PETE, RETI |
|  | D | 52 | 93954 | *ycf2* | 93990 | *ycf2* | ALL |
|  | P | 50 | 76784 | *psbT/psbN* | 76784 | *psbT/psbN* | PETE, RETI |
|  | D | 42 | 101025 | *rps12_3end/* *trnV-GAC* | 122961 | *ndhA intron* | ALL |
|  | D | 42 | 93964 | *ycf2* | 94000 | *ycf2* | ALL |
|  | D | 42 | 45585 | *ycf3 intron* | 122960 | *ndhA intron* | ALL |
|  | D | 42 | 93942 | *ycf2* | 93996 | *ycf2* | ASSA, PETE, RETI |
|  | D | 39 | 45588 | *ycf3 intron* | 101027 | *rps12_3end/trnV-GAC* | ALL |
|  | P | 30 | 9030 | *psbI/trnS-GCU, trnS-GCU* | 47321 | *trnS-GGA,* *trnS-GGA/rps4* | ALL |
|  | D | 38 | 60783 | *accD/psaI* | 60799 | *accD/psaI* | ASSA, PUBI, RETI |
|  | D | 34 | 93954 | *ycf2* | 94008 | *ycf2* | ALL |
|  | D | 30 | 56764 | *atpB/rbcL* | 56768 | *atpB/rbcL* | PETE |
|  | D | 35 | 40523 | *psaB* | 42747 | *psaA* | ALL |
|  | R | 34 | 56764 | *atpB/rbcL* | 56771 | *atpB/rbcL* | PETE |
|  | R | 31 | 56767 | *atpB/rbcL* | 56767 | *atpB/rbcL* | PETE |
|  | D | 33 | 56767 | *atpB/rbcL* | 83105 | *rps8/rpl14* | PETE |
|  | P | 30 | 14186 | *atpF/atpH* | 14186 | *atpF/atpH* | ALL |
|  | D | 30 | 56767 | *atpB/rbcL* | 56768 | *atpB/rbcL* | PETE |
|  | R | 30 | 56767 | *atpB/rbcL* | 56767 | *atpB/rbcL* | PETE |
|  | R | 30 | 56768 | *atpB/rbcL* | 56768 | *atpB/rbcL* | PETE |
|  | D | 30 | 56769 | *atpB/rbcL* | 56771 | *atpB/rbcL* | PETE |
|  | D | 32 | 9028 | *psbI/trnS-GCU, trnS-GCU* | 37367 | *psbC/trnS-UGA, trnS-UGA* | ALL |
|  | R | 32 | 56763 | *atpB/rbcL* | 56767 | *atpB/rbcL* | PETE |
|  | P | 31 | 38688 | *trnfM-CAU/rps14* | 38688 | *trnfM-CAU/rps14* | ALL |
|  | D | 31 | 56764 | *atpB/rbcL* | 56767 | *atpB/rbcL* | PETE |
|  | R | 31 | 56765 | *atpB/rbcL* | 56767 | *atpB/rbcL* | PETE |
|  | R | 31 | 56766 | *atpB/rbcL* | 56771 | *atpB/rbcL* | PETE |
|  | R | 31 | 56767 | *atpB/rbcL* | 56769 | *atpB/rbcL* | PETE |
|  | R | 31 | 56767 | *atpB/rbcL* | 56774 | *atpB/rbcL* | PETE |
|  | R | 31 | 56767 | *atpB/rbcL* | 56775 | *atpB/rbcL* | PETE |
|  | P | 30 | 37369 | *psbC/trnS-UGA, trnS-UGA* | 47321 | *trnS-GGA,* *trnS-GGA/rps4* | ALL |
|  | D | 30 | 45600 | *ycf3 intron* | 101039 | *rps12_3end/trnV-GAC* | ALL |
|  | R | 30 | 56764 | *atpB/rbcL* | 56767 | *atpB/rbcL* | PETE |
|  | R | 30 | 56770 | *atpB/rbcL* | 56771 | *atpB/rbcL* | PETE |
|  | D | 30 | 91506 | *ycf2* | 91548 | *ycf2* | ALL |
| **PUBI** | D | 82 | 93916 | *ycf2* | 93934 | *ycf2* | ALL |
|  | D | 70 | 93928 | *ycf2* | 93946 | *ycf2* | ALL |
|  | D | 60 | 93938 | *ycf2* | 93956 | *ycf2* | ALL |
|  | D | 56 | 93920 | *ycf2* | 93956 | *ycf2* | Single |
|  | D | 52 | 93928 | *ycf2* | 93964 | *ycf2* | ALL |
|  | P | 42 | 79209 | *petD intron* | 79209 | *petD intron* | ASSA |
|  | D | 42 | 100999 | *rps12_3end/trnV-GAC* | 122920 | *ndhA intron* | ALL |
|  | P | 45 | 76763 | *psbT/psbN* | 76763 | *psbT/* *psbN* | ASSA |
|  | D | 42 | 93938 | *ycf2* | 93974 | *ycf2* | ALL |
|  | D | 42 | 45592 | *ycf3 intron* | 122919 | *ndhA intron* | ALL |
|  | D | 39 | 45595 | *ycf3 intron* | 101001 | *rps12_3end/trnV-GAC* | ALL |
|  | P | 30 | 9033 | *psbI/trnS-GCU, trnS-GCU* | 47309 | *trnS-GGA, trnS-GGA/rps4* | ALL |
|  | D | 38 | 60760 | *accD/psaI* | 60776 | *accD/psaI* | ASSA, OLEI, RETI |
|  | D | 38 | 93920 | *ycf2* | 93974 | *ycf2* | Single |
|  | D | 35 | 40529 | *psaB* | 42753 | *psaA* | ALL |
|  | D | 34 | 93928 | *ycf2* | 93982 | *ycf2* | ALL |
|  | P | 30 | 14188 | *atpF/atpH* | 14188 | *atpF/atpH* | ALL |
|  | D | 32 | 9031 | *psbI/trnS-GCU, trnS-GCU* | 37372 | *psbC/trnS-UGA, trnS-UGA* | ALL |
|  | P | 31 | 38694 | *trnfM-CAU/rps14* | 38694 | *trnfM-CAU/rps14* | ALL |
|  | P | 30 | 37374 | *psbC/trnS-UGA, trnS-UGA* | 47309 | *trnS-GGA,* *trnS-GGA/rps4* | ALL |
|  | D | 30 | 45607 | *ycf3 intron* | 101013 | *rps12_3end/trnV-GAC* | ALL |
|  | D | 30 | 91480 | *ycf2* | 91522 | *ycf2* | ALL |
| **PETE** | D | 82 | 93926 | *ycf2* | 93944 | *ycf2* | ALL |
|  | D | 70 | 93938 | *ycf2* | 93956 | *ycf2* | ALL |
|  | D | 60 | 93948 | *ycf2* | 93966 | *ycf2* | ALL |
|  | D | 60 | 93926 | *ycf2* | 93962 | *ycf2* | ASSA, OLEI, RETI |
|  | P | 46 | 79217 | *petD intron* | 79217 | *petD intron* | OLEI, RETI |
|  | D | 52 | 93938 | *ycf2* | 93974 | *ycf2* | ALL |
|  | P | 50 | 76774 | *psbT/psbN* | 76774 | *psbT/psbN* | OLEI, RETI |
|  | D | 42 | 101009 | *rps12_3end/trnV-GAC* | 122940 | *ndhA intron* | ALL |
|  | D | 42 | 93948 | *ycf2* | 93984 | *ycf2* | ALL |
|  | D | 42 | 45601 | *ycf3 intron* | 122939 | *ndhA intron* | ALL |
|  | D | 42 | 93926 | *ycf2* | 93980 | *ycf2* | ASSA, OLEI, RETI |
|  | D | 39 | 45604 | *ycf3 intron* | 101011 | *rps12_3end/trnV-GAC* | ALL |
|  | P | 30 | 9031 | *psbI/trnS-GCU, trnS-GCU* | 47345 | *trnS-GGA,* *trnS-GGA/rps4* | ALL |
|  | D | 33 | 56765 | *atpB/rbcL* | 83089 | *rps8/rpl14* | OLEI |
|  | D | 37 | 60777 | *accD/psaI* | 60793 | *accD/psaI* | Single |
|  | D | 34 | 93938 | *ycf2* | 93992 | *ycf2* | ALL |
|  | D | 30 | 56762 | *atpB/rbcL* | 56766 | *atpB/rbcL* | OLEI |
|  | D | 35 | 40538 | *psaB* | 42762 | *psaA* | ALL |
|  | R | 35 | 56765 | *atpB/rbcL* | 83083 | *rps8/rpl14* | Single |
|  | R | 34 | 56762 | *atpB/rbcL* | 56769 | *atpB/rbcL* | OLEI |
|  | R | 31 | 56765 | *atpB/rbcL* | 56765 | *atpB/rbcL* | OLEI |
|  | P | 30 | 14186 | *atpF/atpH* | 14186 | *atpF/atpH* | ALL |
|  | D | 30 | 56762 | *atpB/rbcL* | 83090 | *rps8/rpl14* | Single |
|  | D | 30 | 56765 | *atpB/rbcL* | 56766 | *atpB/rbcL* | OLEI |
|  | R | 30 | 56765 | *atpB/rbcL* | 56765 | *atpB/rbcL* | OLEI |
|  | R | 30 | 56766 | *atpB/rbcL* | 56766 | *atpB/rbcL* | OLEI |
|  | D | 30 | 56767 | *atpB/rbcL* | 56769 | *atpB/rbcL* | OLEI |
|  | D | 32 | 9029 | *psbI/trnS-GCU, trnS-GCU* | 37381 | *psbC/trnS-UGA, trnS-UGA* | ALL |
|  | R | 32 | 56761 | *atpB/rbcL* | 56765 | *atpB/rbcL* | OLEI |
|  | D | 31 | 13909 | *atpF/atpH* | 56775 | *atpB/rbcL* | Single |
|  | P | 31 | 38703 | *trnfM-CAU/rps14* | 38703 | *trnfM-CAU/rps14* | ALL |
|  | D | 31 | 56762 | *atpB/rbcL* | 56765 | *atpB/rbcL* | OLEI |
|  | R | 31 | 56763 | *atpB/rbcL* | 56765 | *atpB/rbcL* | OLEI |
|  | R | 31 | 56764 | *atpB/rbcL* | 56769 | *atpB/rbcL* | OLEI |
|  | R | 31 | 56765 | *atpB/rbcL* | 56767 | *atpB/rbcL* | OLEI |
|  | R | 31 | 56765 | *atpB/rbcL* | 56772 | *atpB/rbcL* | OLEI |
|  | R | 31 | 56765 | *atpB/rbcL* | 56773 | *atpB/rbcL* | OLEI |
|  | R | 31 | 56765 | *atpB/rbcL* | 83089 | *rps8/rpl14* | Single |
|  | R | 31 | 56765 | *atpB/rbcL* | 83088 | *rps8/rpl14* | Single |
|  | D | 31 | 56765 | *atpB/rbcL* | 83087 | *rps8/rpl14* | Single |
|  | D | 31 | 56765 | *atpB/rbcL* | 83088 | *rps8/rpl14* | Single |
|  | R | 31 | 56767 | *atpB/rbcL* | 83091 | *rps8/rpl14* | Single |
|  | P | 30 | 37383 | *psbC/trnS-UGA, trnS-UGA* | 47345 | *trnS-GGA,* *trnS-GGA/rps4* | ALL |
|  | D | 30 | 45616 | *ycf3 intron* | 101023 | *rps12_3end/trnV-GAC* | ALL |
|  | R | 30 | 56762 | *atpB/rbcL* | 56765 | *atpB/rbcL* | OLEI |
|  | F | 30 | 56765 | *atpB/rbcL* | 83090 | *rps8/rpl14* | Single |
|  | R | 30 | 56765 | *atpB/rbcL* | 83087 | *rps8/rpl14* | Single |
|  | R | 30 | 56766 | *atpB/rbcL* | 83090 | *rps8/rpl14* | Single |
|  | D | 30 | 56766 | *atpB/rbcL* | 83087 | *rps8/rpl14* | Single |
|  | R | 30 | 56768 | *atpB/rbcL* | 56769 | *atpB/rbcL* | OLEI |
|  | D | 30 | 91490 | *ycf2* | 91532 | *ycf2* | ALL |
| **RETI** | D | 82 | 93872 | *ycf2* | 93890 | *ycf2* | ALL |
|  | D | 70 | 93884 | *ycf2* | 93902 | *ycf2* | ALL |
|  | D | 60 | 93894 | *ycf2* | 93912 | *ycf2* | ALL |
|  | D | 60 | 93876 | *ycf2* | 93912 | *ycf2* | ASSA, OLEI, PETE |
|  | P | 46 | 79165 | *petD intron* | 79165 | *petD intron* | OLEI, PETE |
|  | D | 52 | 93884 | *ycf2* | 93920 | *ycf2* | ALL |
|  | P | 50 | 76716 | *psbT/psbN* | 76716 | *psbT/psbN* | OLEI, PETE |
|  | D | 42 | 100955 | *rps12_3end/trnV-GAC* | 122807 | *ndhA intron* | ALL |
|  | D | 42 | 93894 | *ycf2* | 93930 | *ycf2* | ALL |
|  | D | 42 | 45556 | *ycf3 intron* | 122807 | *ndhA intron* | ALL |
|  | D | 42 | 93872 | *ycf2* | 93926 | *ycf2* | ASSA, OLEI, PETE |
|  | D | 39 | 45559 | *ycf3 intron* | 100957 | *rps12_3end/trnV-GAC* | ALL |
|  | P | 30 | 9043 | *psbI/trnS-GCU, trnS-GCU* | 47292 | *trnS-GGA, trnS-GGA/rps4* | ALL |
|  | D | 38 | 60744 | *accD/psaI* | 60760 | *accD/psaI* | ASSA, OLEI, PUBI |
|  | D | 34 | 93884 | *ycf2* | 93938 | *ycf2* | ALL |
|  | D | 35 | 40522 | *psaB* | 42746 | *psaA* | ALL |
|  | P | 30 | 14187 | *atpF/atpH* | 14187 | *atpF/atpH* | ALL |
|  | D | 32 | 9041 | *psbI/trnS-GCU, trnS-GCU* | 37364 | *psbC/trnS-UGA, trnS-UGA* | ALL |
|  | P | 31 | 38687 | *trnfM-CAU/rps14* | 38687 | *trnfM-CAU/rps14* | ALL |
|  | P | 30 | 37366 | *psbC/trnS-UGA, trnS-UGA* | 47292 | *trnS-GGA,* *trnS-GGA/rps4* | ALL |
|  | D | 30 | 45571 | *ycf3 intron* | 100969 | *rps12_3end/trnV-GAC* | ALL |
|  | D | 30 | 91436 | *ycf2* | 91478 | *ycf2* | ALL |

ASSA, *C. sinensis* var. *assamica*; OLEI, *C. oleifera*; PUBI, *C. pubicosta*; PETE, *C. petelotii*; RETI, *C. reticulata.*

D, direct repeat; R, reverse repeat; P, palindromic repeat.
