# Supplementary material for: Thirteen Camellia chloroplast genome sequences determined by high-throughput sequencing: genome structure and phylogenetic relationships
Source: BMC Evol Biol. 2014 Jul 7;14:151. doi: 10.1186/1471-2148-14-151 (PMC4105164; doi:10.1186/1471-2148-14-151)
Supplement: Additional file 5: Table S4 — SSRs characterized in the five Camellia chloroplast genomes. [file 1471-2148-14-151-S5.doc]

**Table S3.** Distribution of simple sequence repeats (SSRs) loci in the five *Camellia* chloroplast genomes

| **Genomes** | **Repeat unit** | **No. repeat unit** | **No. SSR** | **Position A** | **Position B** | **Region** | **Location** | **Locus** |
| --- | --- | --- | --- | --- | --- | --- | --- | --- |
| **ASSA** |  |  |  |  |  |  |  |  |
|  | A | 10 | 6 | 5472 | 5481 | LSC | Intron | *rps16* |
|  |  |  |  | 32927 | 32936 | LSC | IGS | *trnE-UUC/trnT-GGU* |
|  |  |  |  | 44186 | 44195 | LSC | IGS | *psaA/ycf3* |
|  |  |  |  | 65313 | 65322 | LSC | IGS | *petA/psbJ* |
|  |  |  |  | 129878 | 129887 | IR | CDS | *ycf1* |
|  |  |  |  | 138399 | 138408 | IR | IGS | *trnI-GAU/trnI-GAU* |
|  | A | 11 | 5 | 17268 | 17278 | LSC | IGS | *rps2/rpoC2* |
|  |  |  |  | 38267 | 38277 | LSC | IGS | *psbZ/trnG-UCC* |
|  |  |  |  | 73301 | 73311 | LSC | Intron | *clpP* |
|  |  |  |  | 110414 | 110424 | IR | IGS | *rrn5/trnR-ACG* |
|  |  |  |  | 117254 | 117264 | SSC | CDS | *ccsA* |
|  | A | 12 | 6 | 8888 | 8899 | LSC | CDS-IGS | *psbI, psbI/trnS-GCU* |
|  |  |  |  | 38500 | 38511 | LSC | IGS | *trnG-UCC/trnfM-CAU* |
|  |  |  |  | 46254 | 46265 | LSC | Intron | *ycf3* |
|  |  |  |  | 49320 | 49331 | LSC | IGS | *trnT-UGU/trnL-UAA* |
|  |  |  |  | 83624 | 83635 | LSC | IGS | *rpl14/rpl16* |
|  |  |  |  | 129345 | 129356 | IR | CDS | *ycf1* |
|  | A | 13 | 4 | 356 | 368 | LSC | IGS | *trnH-GUG/psbA* |
|  |  |  |  | 32592 | 32604 | LSC | IGS | *trnE-UUC/trnT-GGU* |
|  |  |  |  | 37627 | 37639 | LSC | IGS | *trnS-UGA/psbZ* |
|  |  |  |  | 46510 | 46522 | LSC | IGS | *ycf3/trnS-GGA* |
|  | A | 16 | 1 | 12543 | 12558 | LSC | IGS | *atpA/atpF* |
|  | T | 10 | 9 | 9336 | 9345 | LSC | IGS | *trnS-GCU/trnG-GCC* |
|  |  |  |  | 27174 | 27183 | LSC | CDS | *rpoB* |
|  |  |  |  | 56316 | 56325 | LSC | CDS | *atpB* |
|  |  |  |  | 56702 | 56711 | LSC | IGS | *atpB/rbcL* |
|  |  |  |  | 63138 | 63147 | LSC | IGS | *ycf4/cemA* |
|  |  |  |  | 65451 | 65460 | LSC | IGS | *petA/psbJ* |
|  |  |  |  | 80648 | 80657 | LSC | CDS | *rpoA* |
|  |  |  |  | 82570 | 82579 | LSC | IGS | *infA/rps8* |
|  |  |  |  | 105364 | 105373 | IR | Intron | *trnI-GAU* |
|  | T | 11 | 5 | 19474 | 19484 | LSC | CDS | *rpoC2* |
|  |  |  |  | 52619 | 52629 | LSC | CDS | *ndhK* |
|  |  |  |  | 53357 | 53367 | LSC | IGS | *ndhC/trnV-UAC* |
|  |  |  |  | 70969 | 70979 | LSC | IGS | *rps18/rpl20* |
|  |  |  |  | 133348 | 133358 | IR | IGS | *trnR-ACG/rrn5* |
|  | T | 12 | 6 | 13920 | 13931 | LSC | IGS | *atpF/atpH* |
|  |  |  |  | 15448 | 15459 | LSC | IGS | *atpH/atpI* |
|  |  |  |  | 85075 | 85086 | LSC | Intron | *rpl16* |
|  |  |  |  | 115443 | 115454 | SSC | IGS | *ndhF/rpl32* |
|  |  |  |  | 127426 | 127437 | IR | CDS | *ycf1* |
|  |  |  |  | 129041 | 129052 | IR | CDS | *ycf1* |
|  | T | 13 | 2 | 8744 | 8756 | LSC | IGS | *psbK/psbI* |
|  |  |  |  | 58968 | 58980 | LSC | IGS | *rbcL/accD* |
|  | T | 14 | 3 | 15052 | 15065 | LSC | IGS | *atpH/atpI* |
|  |  |  |  | 56752 | 56765 | LSC | IGS | *atpB/rbcL* |
|  |  |  |  | 121453 | 121466 | SSC | IGS | *ndhG/ndhI* |
|  | T | 15 | 1 | 60748 | 60762 | LSC | IGS | *accD/psaI* |
|  | T | 16 | 1 | 83095 | 83110 | LSC | IGS | *rps8/rpl14* |
|  | T | 17 | 2 | 33230 | 33246 | LSC | IGS | *trnT-GGU/ psbD* |
|  |  |  |  | 130259 | 130275 | IR | CDS | *ycf1* |
|  | AAAAAG | 3 | 1 | 132941 | 132958 | IR | IGS | *trnN-GUU/trnR-ACG* |
|  | CTTTTT | 3 | 1 | 110814 | 110831 | IR | IGS | *trnR-ACG/ trnN-GUU* |
| **Total** |  |  | **53** |  |  |  |  |  |
|  |  |  |  |  |  |  |  |  |
| **OLEI** |  |  |  |  |  |  |  |  |
|  | A | 10 | 5 | 3782 | 3791 | LSC | Intron | *trnK-UUU* |
|  |  |  |  | 49344 | 49353 | LSC | IGS | *trnT-UGU/trnL-UAA* |
|  |  |  |  | 65343 | 65352 | LSC | IGS | *petA/psbJ* |
|  |  |  |  | 129905 | 129914 | IR | CDS | *ycf1* |
|  |  |  |  | 138423 | 138432 | IR | Intron | *trnI-GAU* |
|  | A | 11 | 7 | 349 | 359 | LSC | IGS | *trnH-GUG/psbA* |
|  |  |  |  | 9413 | 9423 | LSC | IGS | *trnS-GCU/trnG-GCC* |
|  |  |  |  | 32926 | 32936 | LSC | IGS | *trnE-UUC/trnT-GGU* |
|  |  |  |  | 38260 | 38270 | LSC | IGS | *psbZ/trnG-UCC* |
|  |  |  |  | 44179 | 44189 | LSC | IGS | *psaA/ycf3* |
|  |  |  |  | 110447 | 110457 | IR | IGS | *rrn5/trnR-ACG* |
|  |  |  |  | 117277 | 117287 | SSC | CDS | *ccsA* |
|  | A | 12 | 6 | 8888 | 8899 | LSC | CDS-IGS | *psbI, psbI/trnS-GCU* |
|  |  |  |  | 17269 | 17280 | LSC | IGS | *rps2/rpoC2* |
|  |  |  |  | 38493 | 38504 | LSC | IGS | *trnG-UCC/trnfM-CAU* |
|  |  |  |  | 46247 | 46258 | LSC | Intron | *ycf3* |
|  |  |  |  | 83650 | 83661 | LSC | IGS | *rpl14/rpl16* |
|  |  |  |  | 129372 | 129383 | IR | CDS | *ycf1* |
|  | A | 13 | 3 | 32591 | 32603 | LSC | IGS | *trnE-UUC/trnT-GGU* |
|  |  |  |  | 37621 | 37633 | LSC | IGS | *trnS-UGA/psbZ* |
|  |  |  |  | 46503 | 46515 | LSC | IGS | *ycf3/ trnS-GGA* |
|  | A | 16 | 1 | 12540 | 12555 | LSC | IGS | *atpA/atpF* |
|  | T | 10 | 7 | 27176 | 27185 | LSC | CDS | *rpoB* |
|  |  |  |  | 56343 | 56352 | LSC | CDS | *atpB* |
|  |  |  |  | 63168 | 63177 | LSC | IGS | *rps16/trnQ-UUG* |
|  |  |  |  | 80674 | 80683 | LSC | CDS | *rpoA* |
|  |  |  |  | 82596 | 82605 | LSC | IGS | *infA/rps8* |
|  |  |  |  | 105389 | 105398 | IR | Intron | *trnI-GAU* |
|  |  |  |  | 116991 | 117000 | SSC | IGS | *trnL-UAG/ccsA* |
|  | T | 11 | 5 | 19476 | 19486 | LSC | CDS | *rpoC2* |
|  |  |  |  | 52646 | 52656 | LSC | CDS | *ndhK* |
|  |  |  |  | 53384 | 53394 | LSC | IGS | *ndhC/ trnV-UAC* |
|  |  |  |  | 85101 | 85111 | LSC | Intron | *rpl16* |
|  |  |  |  | 133372 | 133382 | IR | IGS | *trnR-ACG/rrn5* |
|  | T | 12 | 7 | 13917 | 13928 | LSC | IGS | *atpF/atpH* |
|  |  |  |  | 15049 | 15060 | LSC | IGS | *atpH/atpI* |
|  |  |  |  | 15449 | 15460 | LSC | IGS | *atpH/atpI* |
|  |  |  |  | 58994 | 59005 | LSC | IGS | *rbcL/accD* |
|  |  |  |  | 121476 | 121487 | SSC | IGS | *ndhG/ndhI* |
|  |  |  |  | 127453 | 127464 | IR | CDS | *ycf1* |
|  |  |  |  | 129068 | 129079 | IR | CDS | *ycf1* |
|  | T | 13 | 2 | 73309 | 73321 | LSC | Intron | *clpP* |
|  |  |  |  | 115465 | 115477 | SSC | IGS | *ndhF/rpl32* |
|  | T | 14 | 1 | 8742 | 8755 | LSC | IGS | *psbK/psbI* |
|  | T | 15 | 1 | 60774 | 60788 | LSC | IGS | *accD/psaI* |
|  | T | 16 | 1 | 83121 | 83136 | LSC | IGS | *rps8/rpl14* |
|  | T | 17 | 2 | 33230 | 33246 | LSC | IGS | *trnT-GGU/psbD* |
|  |  |  |  | 130286 | 130302 | IR | CDS | *ycf1* |
|  | T | 27 | 1 | 56772 | 56798 | LSC | IGS | *atpB/rbcL* |
|  | AAAAAG | 3 | 1 | 132968 | 132985 | IR | IGS | *trnN-GUU/trnR-ACG* |
|  | CTTTTT | 3 | 1 | 110836 | 110853 | IR | IGS | *trnR-ACG/ trnN-GUU* |
| **Total** |  |  | **51** |  |  |  |  |  |
|  |  |  |  |  |  |  |  |  |
| **PUBI** |  |  |  |  |  |  |  |  |
|  | A | 10 | 6 | 32925 | 32934 | LSC | IGS | *trnE-UUC/trnT-GGU* |
|  |  |  |  | 65315 | 65324 | LSC | IGS | *petA/psbJ* |
|  |  |  |  | 83625 | 83634 | LSC | IGS | *rpl14/rpl16* |
|  |  |  |  | 115027 | 115036 | SSC | IGS | *ndhF/rpl32* |
|  |  |  |  | 129852 | 129861 | IR | CDS | *ycf1* |
|  |  |  |  | 138367 | 138376 | IR | Intron | *trnI-GAU* |
|  | A | 11 | 5 | 17267 | 17277 | LSC | IGS | *rps2/rpoC2* |
|  |  |  |  | 38266 | 38276 | LSC | IGS | *psbZ/trnG-UCC* |
|  |  |  |  | 73302 | 73312 | LSC | Intron | *clpP* |
|  |  |  |  | 110400 | 110410 | IR | IGS | *rrn5/trnR-ACG* |
|  |  |  |  | 117235 | 117245 | SSC | CDS | *ccsA* |
|  | A | 12 | 7 | 8891 | 8902 | LSC | CDS-IGS | *psbI, psbI/trnS-GCU* |
|  |  |  |  | 32591 | 32602 | LSC | IGS | *trnE-UUC/ trnT-GGU* |
|  |  |  |  | 38499 | 38510 | LSC | IGS | *trnG-UCC/trnfM-CAU* |
|  |  |  |  | 44185 | 44196 | LSC | IGS | *psaA/ycf3* |
|  |  |  |  | 46254 | 46265 | LSC | Intron | *ycf3* |
|  |  |  |  | 49326 | 49337 | LSC | IGS | *trnT-UGU/trnL-UAA* |
|  |  |  |  | 129319 | 129330 | IR | CDS | *ycf1* |
|  | A | 13 | 2 | 37626 | 37638 | LSC | IGS | *trnS-UGA/psbZ* |
|  |  |  |  | 46510 | 46522 | LSC | IGS | *ycf3/trnS-GGA* |
|  | A | 14 | 1 | 356 | 369 | LSC | IGS | *trnH-GUG/psbA* |
|  | A | 16 | 1 | 12542 | 12557 | LSC | IGS | *atpA/atpF* |
|  | T | 10 | 7 | 8749 | 8758 | LSC | IGS | *psbK/psbI* |
|  |  |  |  | 27173 | 27182 | LSC | CDS | *rpoB* |
|  |  |  |  | 56321 | 56330 | LSC | CDS | *atpB* |
|  |  |  |  | 65453 | 65462 | LSC | IGS | *petA/psbJ* |
|  |  |  |  | 80649 | 80658 | LSC | CDS | *rpoA* |
|  |  |  |  | 82571 | 82580 | LSC | IGS | *infA/rps8* |
|  |  |  |  | 105350 | 105359 | IR | Intron | *trnI-GAU* |
|  | T | 11 | 5 | 19473 | 19483 | LSC | CDS | *rpoC2* |
|  |  |  |  | 52625 | 52635 | LSC | CDS | *ndhK* |
|  |  |  |  | 53362 | 53372 | LSC | IGS | *ndhC/trnV-UAC* |
|  |  |  |  | 70970 | 70980 | LSC | IGS | *rps18/rpl20* |
|  |  |  |  | 133316 | 133326 | IR | IGS | *trnR-ACG/rrn5* |
|  | T | 12 | 6 | 13919 | 13930 | LSC | IGS | *atpF/atpH* |
|  |  |  |  | 15447 | 15458 | LSC | IGS | *atpH/atpI* |
|  |  |  |  | 85074 | 85085 | LSC | Intron | *rpl16* |
|  |  |  |  | 115424 | 115435 | SSC | IGS | *ndhF/rpl32* |
|  |  |  |  | 127400 | 127411 | IR | CDS | *ycf1* |
|  |  |  |  | 129015 | 129026 | IR | CDS | *ycf1* |
|  | T | 13 | 1 | 58972 | 58984 | LSC | IGS | *rbcL/accD* |
|  | T | 14 | 3 | 15051 | 15064 | LSC | IGS | *atpH/atpI* |
|  |  |  |  | 60752 | 60765 | LSC | IGS | *accD/psaI* |
|  |  |  |  | 121433 | 121446 | SSC | IGS | *ndhG/ndhI* |
|  | T | 16 | 1 | 83096 | 83111 | LSC | IGS | *rps8/rpl14* |
|  | T | 17 | 2 | 33228 | 33244 | LSC | IGS | *trnT-GGU/psbD* |
|  |  |  |  | 130233 | 130249 | IR | CDS | *ycf1* |
|  | T | 21 | 1 | 56756 | 56776 | LSC | IGS | *atpB/rbcL* |
|  | AAAAAG | 3 | 1 | 132909 | 132926 | IR | IGS | *trnN-GUU/trnR-ACG* |
|  | CTTTTT | 3 | 1 | 110800 | 110817 | IR | IGS | *trnR-ACG/trnN-GUU* |
| **Total** |  |  | **50** |  |  |  |  |  |
|  |  |  |  |  |  |  |  |  |
| **PETE** |  |  |  |  |  |  |  |  |
|  | A | 10 | 7 | 3784 | 3793 | LSC | Intron | *trnK-UUU* |
|  |  |  |  | 5476 | 5485 | LSC | Intron | *rps16* |
|  |  |  |  | 8891 | 8900 | LSC | IGS | *psbI/trnS-GCU* |
|  |  |  |  | 9414 | 9423 | LSC | IGS | *trnS-GCU/trnG-GCC* |
|  |  |  |  | 65337 | 65346 | LSC | IGS | *petA/psbJ* |
|  |  |  |  | 129881 | 129890 | IR | CDS | *ycf1* |
|  |  |  |  | 138399 | 138408 | IR | Intron | *trnI-GAU* |
|  | A | 11 | 7 | 356 | 366 | LSC | IGS | *trnH-GUG/psbA* |
|  |  |  |  | 32932 | 32942 | LSC | IGS | *trnE-UUC/trnT-GGU* |
|  |  |  |  | 38274 | 38284 | LSC | IGS | *psbZ/trnG-UCC* |
|  |  |  |  | 44194 | 44204 | LSC | IGS | *psaA/ycf3* |
|  |  |  |  | 83635 | 83645 | LSC | IGS | *rpl14/rpl16* |
|  |  |  |  | 110431 | 110441 | IR | IGS | *rrn5/ trnR-ACG* |
|  |  |  |  | 117259 | 117269 | SSC | CDS | *ccsA* |
|  | A | 12 | 5 | 17271 | 17282 | LSC | IGS | *rps2/rpoC2* |
|  |  |  |  | 37635 | 37646 | LSC | IGS | *trnS-UGA/psbZ* |
|  |  |  |  | 46263 | 46274 | LSC | Intron | *ycf3* |
|  |  |  |  | 49347 | 49358 | LSC | IGS | *trnT-UGU/trnL-UAA* |
|  |  |  |  | 129348 | 129359 | IR | CDS | *ycf1* |
|  | A | 13 | 3 | 32597 | 32609 | LSC | IGS | *trnE-UUC/trnT-GGU* |
|  |  |  |  | 38507 | 38519 | LSC | IGS | *trnG-UCC/trnfM-CAU* |
|  |  |  |  | 46519 | 46531 | LSC | IGS | *ycf3/trnS-GGA* |
|  | A | 16 | 1 | 12540 | 12555 | LSC | IGS | *atpA/atpF* |
|  | T | 10 | 10 | 27178 | 27187 | LSC | CDS | *rpoB* |
|  |  |  |  | 33596 | 33605 | LSC | IGS | *trnT-GGU/psbD* |
|  |  |  |  | 48945 | 48954 | LSC | IGS | *trnT-UGU/trnL-UAA* |
|  |  |  |  | 56342 | 56351 | LSC | CDS | *atpB* |
|  |  |  |  | 61523 | 61532 | LSC | IGS | *psaI/ycf4* |
|  |  |  |  | 70992 | 71001 | LSC | IGS | *rps18/rpl20* |
|  |  |  |  | 73299 | 73308 | LSC | Intron | *clpP* |
|  |  |  |  | 80658 | 80667 | LSC | CDS | *rpoA* |
|  |  |  |  | 82580 | 82589 | LSC | IGS | *infA/rps8* |
|  |  |  |  | 105373 | 105382 | IR | Intron | *trnI-GAU* |
|  | T | 11 | 6 | 19478 | 19488 | LSC | CDS | *rpoC2* |
|  |  |  |  | 52646 | 52656 | LSC | CDS | *ndhK* |
|  |  |  |  | 53383 | 53393 | LSC | IGS | *ndhC/trnV-UAC* |
|  |  |  |  | 65790 | 65800 | LSC | IGS | *petA/psbJ* |
|  |  |  |  | 85085 | 85095 | LSC | Intron | *rpl16* |
|  |  |  |  | 133340 | 13262 | IR | IGS | *trnR-ACG/rrn5* |
|  | T | 12 | 5 | 8747 | 8758 | LSC | IGS | *psbK/psbI* |
|  |  |  |  | 15451 | 15462 | LSC | IGS | *atpH/atpI* |
|  |  |  |  | 115449 | 115460 | SSC | IGS | *ndhF/rpl32* |
|  |  |  |  | 127429 | 127440 | IR | CDS | *ycf1* |
|  |  |  |  | 129044 | 129055 | IR | CDS | *ycf1* |
|  | T | 13 | 1 | 58992 | 59004 | LSC | IGS | *rbcL/accD* |
|  | T | 14 | 2 | 15049 | 15062 | LSC | IGS | *atpH/atpI* |
|  |  |  |  | 121458 | 121471 | SSC | IGS | *ndhG/ndhI* |
|  | T | 15 | 1 | 60772 | 60786 | LSC | IGS | *accD/psaI* |
|  | T | 17 | 2 | 33236 | 33252 | LSC | IGS | *trnT-GGU/psbD* |
|  |  |  |  | 130262 | 130278 | IR | CDS | *ycf1* |
|  | T | 19 | 2 | 13910 | 13928 | LSC | IGS | *atpF/atpH* |
|  |  |  |  | 83102 | 83120 | LSC | IGS | *rps8/rpl14* |
|  | T | 27 | 1 | 56770 | 56796 | LSC | IGS | *atpB/rbcL* |
|  | AAAAAG | 3 | 1 | 132944 | 132961 | IR | IGS | *trnN-GUU/trnR-ACG* |
|  | CTTTTT | 3 | 1 | 110820 | 110837 | IR | IGS | *trnR-ACG/trnN-GUU* |
| **Total** |  |  | **55** |  |  |  |  |  |
|  |  |  |  |  |  |  |  |  |
| **REIT** |  |  |  |  |  |  |  |  |
|  | A | 10 | 6 | 3791 | 3800 | LSC | Intron | *trnK-UUU* |
|  |  |  |  | 4456 | 4465 | LSC | IGS | *trnK-UUU/rps16* |
|  |  |  |  | 44178 | 44187 | LSC | IGS | *psaA/ycf3* |
|  |  |  |  | 65293 | 65302 | LSC | IGS | *petA/psbJ* |
|  |  |  |  | 129741 | 129750 | IR | CDS | *ycf1* |
|  |  |  |  | 138258 | 138267 | IR | Intron | *trnI-GAU* |
|  | A | 11 | 7 | 8902 | 8912 | LSC | CDS-IGS | *psbI, psbI/trnS-GCU* |
|  |  |  |  | 17261 | 17271 | LSC | IGS | *rps2/rpoC2* |
|  |  |  |  | 32914 | 32924 | LSC | IGS | *trnE-UUC/trnT-GGU* |
|  |  |  |  | 38258 | 38268 | LSC | IGS | *psbZ/trnG-UCC* |
|  |  |  |  | 83581 | 83591 | LSC | IGS | *rpl14/rpl16* |
|  |  |  |  | 110362 | 110372 | IR | IGS | *rrn5/trnR-ACG* |
|  |  |  |  | 117122 | 117132 | SSC | CDS | *ccsA* |
|  | A | 12 | 3 | 46218 | 46229 | LSC | Intron | *ycf3* |
|  |  |  |  | 49314 | 49325 | LSC | IGS | *trnT-UGU/trnL-UAA* |
|  |  |  |  | 129208 | 129219 | IR | CDS | *ycf1* |
|  | A | 13 | 5 | 356 | 368 | LSC | IGS | *trnH-GUG/psbA* |
|  |  |  |  | 32579 | 32591 | LSC | IGS | *trnE-UUC/trnT-GGU* |
|  |  |  |  | 37618 | 37630 | LSC | IGS | *trnS-UGA/psbZ* |
|  |  |  |  | 38491 | 38503 | LSC | IGS | *trnG-UCC/trnfM-CAU* |
|  |  |  |  | 46474 | 46486 | LSC | IGS | *ycf3/trnS-GGA* |
|  | A | 16 | 1 | 12546 | 12561 | LSC | IGS | *atpA/atpF* |
|  | T | 10 | 11 | 27161 | 27170 | LSC | CDS | *rpoB* |
|  |  |  |  | 37218 | 37227 | LSC | IGS | *psbC/trnS-UGA* |
|  |  |  |  | 49971 | 49980 | LSC | Intron | *trnL-UAA* |
|  |  |  |  | 53462 | 53471 | LSC | Intron | *trnV-UAC* |
|  |  |  |  | 56307 | 56316 | LSC | CDS | *atpB* |
|  |  |  |  | 65431 | 65440 | LSC | IGS | *petA/psbJ* |
|  |  |  |  | 70937 | 70946 | LSC | IGS | *rps18/rpl20* |
|  |  |  |  | 80605 | 80614 | LSC | CDS | *rpoA* |
|  |  |  |  | 82527 | 82536 | LSC | IGS | *infA/rps8* |
|  |  |  |  | 105304 | 105313 | IR | Intron | *trnI-GAU* |
|  |  |  |  | 117949 | 117958 | SSC | IGS | *ccsA/ndhD* |
|  | T | 11 | 6 | 8759 | 8769 | LSC | IGS | *psbK/psbI* |
|  |  |  |  | 19461 | 19471 | LSC | CDS | *rpoC2* |
|  |  |  |  | 52609 | 52619 | LSC | CDS | *ndhK* |
|  |  |  |  | 58957 | 58967 | LSC | IGS | *rbcL/accD* |
|  |  |  |  | 85031 | 85041 | LSC | Intron | *rpl16* |
|  |  |  |  | 133199 | 133209 | IR | IGS | *trnR-ACG/rrn5* |
|  | T | 12 | 3 | 13918 | 13929 | LSC | IGS | *atpF/atpH* |
|  |  |  |  | 127289 | 127300 | IR | CDS | *ycf1* |
|  |  |  |  | 128904 | 128915 | IR | CDS | *ycf1* |
|  | T | 13 | 2 | 15446 | 15458 | LSC | IGS | *atpH/atpI* |
|  |  |  |  | 115309 | 115321 | SSC | IGS | *ndhF/rpl32* |
|  | T | 14 | 3 | 15044 | 15057 | LSC | IGS | *atpH/atpI* |
|  |  |  |  | 65747 | 65760 | LSC | IGS | *petA/psbJ* |
|  |  |  |  | 121321 | 121334 | SSC | IGS | *ndhG/ndhI* |
|  | T | 15 | 1 | 60735 | 60749 | LSC | IGS | *accD/psaI* |
|  | T | 16 | 2 | 53347 | 53362 | LSC | IGS | *ndhC/trnV-UAC* |
|  |  |  |  | 83052 | 83067 | LSC | IGS | *rps8/rpl14* |
|  | T | 17 | 2 | 33218 | 33234 | LSC | IGS | *trnT-GGU/psbD* |
|  |  |  |  | 130122 | 130138 | IR | CDS | *ycf1* |
|  | T | 21 | 1 | 56741 | 56761 | LSC | IGS | *atpB/rbcL* |
|  | CCCT | 4 | 1 | 110062 | 110077 | IR | IGS | *rrn4.5/rrn5* |
|  | CTTTTT | 3 | 1 | 110762 | 110779 | IR | IGS | *trnR-ACG/trnN-GUU* |
| **Total** |  |  | **55** |  |  |  |  |  |

ASSA, *C. sinensis* var. *assamica*; OLEI, *C. oleifera*; PUBI, *C. pubicosta*; PETE, *C. petelotii*; RETI, *C. reticulata*. IGS, intergenic spacer; CDS, coding sequence.
