# Supplementary material for: Thirteen Camellia chloroplast genome sequences determined by high-throughput sequencing: genome structure and phylogenetic relationships
Source: BMC Evol Biol. 2014 Jul 7;14:151. doi: 10.1186/1471-2148-14-151 (PMC4105164; doi:10.1186/1471-2148-14-151)
Supplement: Additional file 6: Table S5 — The information of candidate markers with the variations more than 1.5%. [file 1471-2148-14-151-S6.doc]

**Table S5.** The information of potential molecular markers with the variations more than 1.5 %.

| **Region** | **Location** | **Aligned length (bp)** | | | |  | **No. of variable sites (indels)** | | | | | **Percentage of variable sites** | | | | |
| --- | --- | --- | --- | --- | --- | --- | --- | --- | --- | --- | --- | --- | --- | --- | --- | --- |
|  |  | ASSA | OLEI | PUBI | PETE | RETI | ASSA | OLEI | PUBI | PETE | RETI | ASSA | OLEI | PUBI | PETE | RETI |
| *trnH-GUG/psbA* | LSC | 382 | 373 | 383 | 380 | 382 | 14(4) | 29(7) | 12(5) | 14(4) | 11(4) | 3.66 | 7.77 | 3.13 | 3.68 | 2.88 |
| *psbK/psbI* | LSC | 396 | 396 | 394 | 396 | 395 | 9(2) | 15(2) | 8(4) | 6(2) | 8(4) | 2.27 | 3.79 | 2.03 | 1.52 | 2.03 |
| *trnS-GCU/trnG-GCC* | LSC | 675 | 672 | 671 | 671 | 670 | 38(11) | 91(7) | 43(8) | 37(7) | 41(5) | 5.63 | 13.54 | 6.41 | 5.51 | 6.12 |
| *trnG-GCC intron* | LSC | 705 | 705 | 705 | 705 | 700 | 24(1) | 24(1) | 18(1) | 15(1) | 27(4) | 3.40 | 3.40 | 2.55 | 2.13 | 3.86 |
| *atpF/atpH* | LSC | 376 | 376 | 376 | 376 | 371 | 9(1) | 8(1) | 12(1) | 17(1) | 14(4) | 2.39 | 2.13 | 3.19 | 4.52 | 3.77 |
| *trnE-UUC/trnT-GGU* | LSC | 773 | 770 | 772 | 774 | 774 | 12(5) | 21(7) | 15(8) | 16(4) | 12(4) | 1.55 | 2.73 | 1.94 | 2.07 | 1.55 |
| *trnS-UGA/psbZ* | LSC | 355 | 355 | 355 | 354 | 355 | 7(1) | 7(1) | 7(1) | 9(4) | 10(1) | 1.97 | 1.97 | 1.97 | 2.54 | 2.82 |
| *psaA/ycf3* | LSC | 788 | 788 | 789 | 789 | 760 | 18(7) | 14(6) | 15(7) | 12(7) | 21(9) | 2.28 | 1.78 | 1.90 | 1.52 | 2.76 |
| *trnT-UGU/trnL-UAA* | LSC | 980 | 990 | 986 | 982 | 991 | 19(10) | 24(10) | 15(9) | 21(10) | 15(7) | 1.94 | 2.42 | 1.52 | 2.14 | 1.51 |
| *trnP-UGG/psaJ* | LSC | 390 | 384 | 390 | 384 | 391 | 14(3) | 7(3) | 14(3) | 7(3) | 26(4) | 3.59 | 1.82 | 3.59 | 1.82 | 6.65 |
| *rps18/rpl20* | LSC | 262 | 254 | 262 | 255 | 255 | 6(6) | 6(6) | 6(6) | 5(5) | 5(5) | 2.29 | 2.36 | 2.29 | 1.96 | 1.96 |
| *petD/rpoA* | LSC | 205 | 205 | 205 | 205 | 204 | 6(1) | 6(1) | 14(1) | 9(1) | 11(4) | 2.93 | 2.93 | 6.83 | 4.39 | 5.39 |
| *ycf15/trnL-CAA* | IR | 370 | 370 | 370 | 370 | 370 | 12(0) | 8(0) | 12(0) | 8(0) | 8(0) | 3.24 | 2.16 | 3.24 | 2.16 | 2.16 |
| *ndhF/rpl32* | SSC | 679 | 680 | 680 | 679 | 681 | 18(3) | 15(5) | 18(6) | 15(3) | 24(5) | 2.65 | 2.21 | 2.65 | 2.21 | 3.52 |
| *ccsA/ndhD* | SSC | 238 | 238 | 237 | 238 | 238 | 6(1) | 5(1) | 8(3) | 7(0) | 8(1) | 2.52 | 2.10 | 3.38 | 2.94 | 3.36 |
